# Exercise modalities and dose for improving arterial stiffness in risk-enriched adults: a systematic review and dose-response network meta-analysis

**DOI:** 10.1186/s12872-026-06208-7

**Published:** 2026-07-09

**Authors:** Shiqi Liu, Yanli Tan, Liuhong Zang

**Affiliations:** Institute of Physical Education, Xinjiang Normal University, Urumqi, Xinjiang 830017 China

**Keywords:** Arterial stiffness, Carotid-femoral pulse wave velocity, Exercise intervention, Cardiovascular risk, Network meta-analysis

## Abstract

**Background:**

Exercise is recommended for cardiovascular prevention, but the comparative effects of different exercise modalities and weekly doses on carotid-femoral pulse wave velocity (cfPWV) in heterogeneous risk-enriched adults with cardiovascular risk-related conditions or characteristics remain uncertain. This systematic review and dose-response network meta-analysis aimed to compare exercise modalities for improving cfPWV and to explore model-derived dose-response patterns and approximate favorable dose regions.

**Methods:**

PubMed, Cochrane Library, Web of Science, Embase, SPORTDiscus, and CINAHL were searched from inception to 22 February 2026 for English-language parallel-group randomized controlled trials reporting cfPWV in heterogeneous risk-enriched adults with cardiovascular risk-related conditions or characteristics. Pairwise meta-analysis, frequentist network meta-analysis, and Bayesian dose-response network meta-analysis were performed.

**Results:**

Thirty-seven studies involving 2,110 participants were included. In direct pairwise analysis, exercise was associated with a reduction in cfPWV compared with control (WMD = -0.65, 95% CI -1.13 to -0.18), but heterogeneity was considerable (I² = 94.4%). Aerobic training and whole-body vibration training showed significant reductions versus control in modality-specific direct comparisons, whereas resistance training, combined training, interval training, and isometric handgrip training did not reach statistical significance. In the network meta-analysis, aerobic training (WMD = -0.65, 95% CI -1.08 to -0.22) and interval training (WMD = -1.07, 95% CI -1.66 to -0.49) were associated with lower cfPWV than control. The dose-response model suggested a nonlinear association, with the lower model-derived crossing region around 200–300 MET·min/week and the lowest fitted estimate around 700–800 MET·min/week at the overall exercise level. However, these values are approximate model-derived ranges, not exact clinical thresholds, and should be interpreted cautiously because of clinical heterogeneity, sparse evidence in some dose regions and exercise nodes, and uncertainty around threshold estimates.

**Conclusions:**

Exercise may reduce cfPWV in heterogeneous risk-enriched adults with cardiovascular risk-related conditions or characteristics, particularly through aerobic and interval-based training in the network synthesis. Direct pairwise evidence supported exercise overall, aerobic training, and whole-body vibration training, but the certainty of evidence was generally low or very low and heterogeneity was considerable. The dose-response findings suggest a potential moderate-dose region with lower model-predicted cfPWV estimates at the overall exercise level, but these estimates should be considered model-derived and hypothesis-generating rather than definitive clinical prescription thresholds. These findings should be interpreted as average effects across diverse populations and exercise prescriptions rather than as uniform expected effects.

**Trial registration:**

PROSPERO CRD420261372601.

**Supplementary Information:**

The online version contains supplementary material available at https://doi.org/10.1186/s12872-026-06208-7.

## Background

Arterial stiffness (AS) is a major marker of vascular aging and an established predictor of future cardiovascular risk. Among the available indices, carotid-femoral pulse wave velocity (cfPWV) is widely recognized as the gold-standard noninvasive measure of central arterial stiffness because it directly reflects aortic stiffness and has clear prognostic value [1, 2]. Previous evidence has shown that each 1 m/s increase in aortic PWV is associated with increased risks of cardiovascular events, cardiovascular mortality, and all-cause mortality [3]. Therefore, identifying effective non-pharmacological strategies to reduce cfPWV is of considerable clinical importance.

This issue is especially relevant in heterogeneous risk-enriched adults with cardiovascular risk-related conditions or characteristics. In the present review, the target population was defined pragmatically as adults with established or widely recognized risk-related conditions or characteristics, including overweight or obesity, diabetes, hypertension or prehypertension, metabolic syndrome, older age, and postmenopausal status. Older age and postmenopausal status were not treated as disease states equivalent to diabetes or hypertension, but as life-stage characteristics associated with increased vascular stiffness and cardiovascular risk. Although these groups are not clinically identical, they share a higher likelihood of adverse vascular remodeling, metabolic or hemodynamic stress, endothelial dysfunction, and accelerated arterial stiffening.

 [4–7]. Therefore, they were considered together as a broad prevention-oriented, risk-enriched adult population, while recognizing that the magnitude of exercise-related changes in cfPWV may differ across subgroups.

Exercise is a cornerstone of cardiovascular prevention and is strongly recommended in current physical activity guidelines [8]. It may improve arterial stiffness through multiple mechanisms, including enhanced endothelial function, favorable arterial remodeling, reduced sympathetic vasoconstrictor tone, and improved hemodynamic regulation [9]. However, the vascular benefits of exercise do not appear to be uniform across exercise modalities. Aerobic training is generally considered beneficial for vascular health, interval training may confer stronger hemodynamic and metabolic stimuli in some populations, whereas resistance-based interventions have shown more heterogeneous findings depending on training intensity, protocol structure, and participant characteristics [10–12].

Several systematic reviews have shown that exercise can improve arterial stiffness, but the existing evidence remains fragmented in ways that limit its value for exercise prescription in adults at cardiovascular disease (CVD) risk. Ashor et al. synthesized multiple exercise modalities across broad adult populations and vascular outcomes, whereas Montero et al. focused specifically on aerobic training in pre- and hypertensive subjects [12, 13]. More recently, Saz-Lara et al. compared exercise modalities using network meta-analysis in the general adult population, and Wu et al. extended this comparative approach to individuals at risk for CVD [14, 15]. Taken together, these reviews support the vascular relevance of exercise, but they do not fully resolve which exercise strategies are most appropriate for prevention-oriented high-risk populations.

Three important gaps remain. First, prior syntheses have often combined clinically heterogeneous populations, either by mixing healthy adults with at-risk adults or by focusing on only one subgroup, which limits direct inference for adults with elevated cardiovascular risk as a broader prevention target [12–14]. Second, outcome definitions have frequently been heterogeneous, with different pulse wave velocity indices or vascular markers considered together, even though carotid-femoral pulse wave velocity (cfPWV) is the reference-standard measure of central arterial stiffness and offers greater clinical specificity [12, 15]. Third, previous reviews have mainly compared exercise modalities at the intervention level, but have rarely quantified weekly exercise dose in a way that can inform cautious clinical interpretation and guide future trial design.

Previous syntheses have established that exercise can influence arterial stiffness, but their clinical interpretability for central arterial stiffness is limited when different PWV measurement sites or converted PWV estimates are combined. The main incremental contribution of this review is its cfPWV-specific design, which preserves the clinical meaning of central arterial stiffness and avoids treating peripheral or mixed PWV indices as interchangeable with central aortic stiffness. Within this focused framework, pairwise meta-analysis, network meta-analysis, GRADE certainty assessment, and exploratory dose-response modeling were integrated to summarize comparative exercise effects in heterogeneous risk-enriched adults.

Accordingly, the present study aimed to synthesize randomized controlled evidence on exercise effects on cfPWV in adults with cardiovascular risk-related conditions or characteristics, compare exercise modalities within a cfPWV-specific network, and explore model-derived dose-response patterns and approximate favorable weekly dose regions.

## Methods

### Registration

This systematic review and network meta-analysis was registered in PROSPERO on 18 February 2026 (registration number: CRD420261372601) and was conducted and reported in accordance with the Preferred Reporting Items for Systematic Reviews and Meta-Analyses extension statement for reporting systematic reviews incorporating network meta-analyses [16] and the Cochrane Handbook for Systematic Reviews of Interventions [17].

### Search strategy

We systematically searched the following electronic databases: PubMed, Cochrane Library, Web of Science, Embase, SPORTDiscus, and CINAHL, from database inception to 22 February 2026, to identify eligible studies. SPORTDiscus and CINAHL were searched via the EBSCOhost platform. Only articles published in English were considered. The search strategy was developed by combining controlled vocabulary terms and free-text terms with Boolean operators and was structured around the following key concepts: exercise interventions, cardiovascular-risk populations, carotid-femoral pulse wave velocity, and randomized controlled trials. The full search strategies for all searched sources are provided in Additional file 1: Tables S1-S5.

### Eligibility criteria

Eligible studies were English-language full-text parallel-group randomized controlled trials that evaluated the effects of exercise training on cfPWV in adults aged ≥ 18 years with cardiovascular risk-related conditions or characteristics. For the purpose of this review, cardiovascular risk-related populations were operationally defined as adults with conditions or life-stage characteristics commonly associated with elevated vascular risk, including overweight or obesity, diabetes, hypertension or prehypertension, metabolic syndrome, older age, and postmenopausal status. Older age and postmenopausal status were treated as life-stage characteristics associated with vascular risk rather than disease states equivalent to diabetes or hypertension. These populations were grouped to reflect a broad prevention-oriented, risk-enriched adult population rather than a single homogeneous disease category. Exercise interventions included aerobic training (AT), resistance training (RT), interval training (IT), combined training (CT), stretching training (ST), isometric handgrip training (IHT), and whole-body vibration training (WBVT). In this review, IT included interval-based aerobic exercise protocols characterized by repeated higher-intensity exercise bouts interspersed with lower-intensity recovery or rest, such as high-intensity interval training, interval walking, and sprint interval training, as described in the original trials. Comparators included non-exercise, usual-care, or no-structured-exercise control conditions, as well as active comparator arms involving another exercise modality when multi-arm exercise trials were available. Studies reporting aortic PWV were also eligible when the measurement clearly corresponded to the carotid-femoral pathway.

Exclusion criteria were: crossover, cluster-randomized, non-randomized, quasi-experimental, and single-arm studies; animal studies; studies involving children or adolescents; acute single-session exercise studies; studies conducted in healthy adults without cardiovascular risk factors; studies in which the independent effect of exercise could not be isolated; studies reporting only baPWV, faPWV, or other local arterial stiffness indices without cfPWV-related data; and studies with insufficient data, duplicate publication, conference abstract, review, letter, or meta-analysis.

### Study selection and data extraction

All records were imported into Zotero 8 for reference management and duplicate removal. Study selection was performed independently by two reviewers (S.L. and Y.T.), who screened titles and abstracts and then assessed the full texts of potentially eligible articles. Disagreements were resolved by discussion, with consultation of a third reviewer (L.Z.) when necessary. Potential companion or overlapping reports were checked by comparing author groups, trial identifiers, recruitment settings or periods, participant characteristics, sample sizes, intervention arms, and outcome reporting. When multiple reports were judged to use the same participant cohort, only the report providing the most complete and directly usable cfPWV data for the present analyses was retained to avoid double counting. Data extraction was also conducted independently by the same two reviewers using a standardised form. The following information was extracted from each eligible study: study reference, country, study design, participant characteristics (sample size, sex, age, and population type), intervention characteristics (exercise modality, duration, frequency, session length, and intensity), comparator characteristics, and outcome characteristics related to cfPWV assessment. Exercise intensity indicators, when reported, were extracted as originally described in each study, including %HRR, %HRmax, %VO2max, %1RM, or other protocol-specific descriptions. The characteristics of the included studies are presented in Additional file 1: Table S6.

### Outcome definition

The primary outcome was the carotid-femoral pulse wave velocity (cfPWV). Studies reporting aortic PWV were also eligible when the measurement clearly corresponded to the carotid-femoral pathway. cfPWV was selected because it is widely considered the gold-standard measure of arterial stiffness [1, 2]. In the present review, cfPWV was extracted and synthesized as a continuous outcome.

### Data synthesis

Effect sizes were synthesized using pre-to-post changes in the experimental and control groups to minimize the influence of baseline differences. When change scores were not directly reported, mean changes were calculated from baseline and post-intervention values. The corresponding standard deviations of change were derived according to the Cochrane Handbook using the formula [17]:

$$\mathrm{SDchange}=\sqrt{\left(\mathrm{SDbaseline}^{2}+\mathrm{SDpost}^{2}-2\times\mathrm{r}\times\mathrm{SDbaseline}\times\mathrm{SDpost}\right)}$$

When the within-group correlation coefficient (r) was not reported, r was assumed to be 0.5 for the primary analysis. Outcomes reported as medians, ranges, or interquartile ranges were converted to means and standard deviations using established methods [18].

### Statistical analyses

#### Pairwise meta-analysis

Pairwise meta-analyses were performed in Stata 17.0 using a random-effects model with the DerSimonian–Laird method. Because cfPWV was reported in a consistent unit across studies, pooled effects were expressed as weighted mean differences (WMDs) with 95% confidence intervals (CIs). Statistical heterogeneity was assessed using the I² statistic, whose values were considered not important (0%–30%), moderate (30%–50%), substantial (50%–75%) or considerable (75%–100%), and corresponding p values were also taken into account [19]. For multi-arm trials included in pairwise meta-analyses, the shared control group was split evenly across the relevant comparisons to avoid double counting.

Sensitivity analyses were conducted using the leave-one-out method and by repeating the pairwise meta-analyses with alternative correlation coefficients of *r* = 0.25 and *r* = 0.75 for imputing change-score SDs, with *r* = 0.50 used as the primary assumption. These correlation-sensitivity analyses focused on the overall exercise-versus-control effect and exercise-modality subgroups. To explore potential sources of heterogeneity, subgroup analyses and meta-regression were conducted for the overall exercise-versus-control comparison, and leave-one-out sensitivity analyses were conducted for the AT versus control and IT versus control comparisons because these direct comparisons showed considerable heterogeneity. The IT versus control leave-one-out analysis was interpreted cautiously because only three studies were available in the direct pairwise forest plot. The presence of publication bias was determined by Egger’s test and visual inspection of the funnel plot.

#### Network meta-analysis

A random-effects network meta-analysis was performed in Stata 17.0 under a frequentist framework to synthesize direct and indirect evidence [20, 21]. Before conducting the network meta-analysis, the plausibility of transitivity was assessed qualitatively by comparing the distributions of prespecified clinical and intervention-related effect modifiers across treatment nodes and major direct comparisons. These effect modifiers included population category, age, BMI, sex distribution, baseline cfPWV, intervention duration, session length, weekly frequency, weekly exercise dose, and medication use where reported. Statistical consistency was assessed separately and was not used to establish transitivity. The geometry of the treatment network was illustrated using a network plot, with node size proportional to sample size and edge thickness proportional to the number of direct comparisons. The consistency of the network was assessed using the inconsistency factor (IF) and its 95% CI, together with node-splitting analysis and the global inconsistency model [21, 22]. When no statistically significant inconsistency was detected (*P* > 0.05), the consistency model was used for the main analysis [22]. SUCRA values were calculated to summarize ranking probabilities [20, 23]. They were used only as supportive and exploratory summaries and were not interpreted as evidence of clinically meaningful superiority; ranking results were considered together with effect estimates, confidence intervals, direct evidence, network sparsity, and GRADE certainty. Comparison-adjusted funnel plots were used to assess potential small-study effects. In addition, an interval plot based on the consistency model was used to present the pooled estimates with 95% confidence intervals and 95% prediction intervals for all pairwise comparisons [21, 24]. Network sensitivity analyses were also conducted using alternative correlation coefficients of *r* = 0.25 and *r* = 0.75 for imputing change-score SDs, with *r* = 0.50 used as the primary assumption. The corresponding league-table estimates and SUCRA rankings were compared across these correlation assumptions.

#### Dose-response analysis

Exercise dose was quantified as weekly energy expenditure and expressed as MET·min/week. The weekly exercise dose was calculated using the following formula:$$\begin{aligned} &\text{assigned MET value}\times\text{session duration}\left(\mathrm{min}\right)\\&\times\text{weekly frequency}\left(\mathrm{sessions/week}\right)\\&=\text{weekly exercise dose}\:(\mathrm{MET} \cdot \mathrm{min/week}) \end{aligned}$$. MET values were derived primarily from exercise intensity indicators reported in the original studies, including heart rate reserve, percentage of maximal heart rate, percentage of maximal oxygen uptake, or rating of perceived exertion. When multiple intensity indicators were available, the indicator most directly linked to aerobic or metabolic intensity was prioritized. When intensity indicators were unavailable, MET values were assigned according to the described exercise modality with reference to the 2024 Compendium of Physical Activities for Adults and relevant exercise-intensity classifications [25–29].

To ensure the connectivity and stability of the dose-response network meta-analysis, the calculated weekly doses were further assigned to predefined discrete dose categories. Based on the distribution of extracted exercise doses in the included trials, the following dose categories were used: 0, 300, 600, 900, 1200, and 1500 MET·min/week. Each exact calculated dose was converted to the nearest predefined dose category, using the midpoint between two adjacent dose categories as the cutoff. For example, an exact dose from 450 to < 750 MET·min/week was categorized as 600 MET·min/week. Because 0 MET·min/week was reserved for control conditions, active exercise doses below 300 MET·min/week were assigned to the lowest active dose category of 300 MET·min/week. These predefined categories were used as analytical dose nodes to preserve dose-network connectivity and model stability, rather than as biologically discrete thresholds or clinically prescriptive cut-points. The exact calculated dose and the rounded dose category used in the dose-response model are reported for each intervention arm in Additional file 1: Table S6.

Control groups were assigned a dose of 0 MET·min/week. This dose-standardization approach was applied uniformly across exercise modalities to improve comparability across trials. MET·min/week was used as a pragmatic common exposure metric rather than as a complete representation of modality-specific mechanical or neuromuscular training load. For resistance and combined training, weekly dose was calculated using the same MET-based formula based on the assigned modality-specific MET value, session duration, and weekly frequency; however, this approach did not capture resistance load, repetitions, sets, rest intervals, or time under tension. For whole-body vibration training, MET·min/week did not capture vibration frequency, amplitude, posture, or exposure pattern. For isometric handgrip training, it did not capture contraction intensity, contraction duration, or duty cycle. Dose estimation is therefore more directly interpretable for aerobic training and interval training, in which session duration and intensity more closely reflect the main exercise exposure. By contrast, for resistance training, combined training, whole-body vibration training, isometric handgrip training, and stretching training, MET·min/week may be less precise because total session duration may not fully capture active contraction time, loading volume, rest intervals, time under tension, muscle groups involved, or modality-specific physiological exposure. Therefore, exercise-type-specific dose-response findings for these modalities were interpreted cautiously and should be regarded as exploratory rather than as definitive modality-specific dose prescriptions.

The dose-response relationship between exercise dose and carotid-femoral pulse wave velocity (cfPWV) was examined using a random-effects Bayesian model-based network meta-analysis (MBNMA) [30]. Before model fitting, the key assumptions of MBNMA were evaluated through network connectivity, consistency, and transitivity [30–32]. Different nonlinear models, including the Emax, restricted cubic spline (using the default knot specification implemented in MBNMAdose), and quadratic models, were compared using the Deviance Information Criterion (DIC) and fitted plots. The quadratic random-effects model was selected as the final analytical model because it showed a slightly lower DIC than the restricted cubic spline model and provided a more parsimonious and interpretable description of the observed nonlinear dose-response pattern [33]. Mean difference (MD) was used as the effect size measure in the dose-response analysis, and 95% credible intervals (CrIs) were used to judge statistical uncertainty; results were considered statistically significant when the 95% CrIs did not include 0 [34]. All dose-response analyses were conducted using the “MBNMAdose” package in R, and the figures were generated using “ggplot2.” Bayesian dose-response models were fitted using the default prior settings implemented in the MBNMAdose/JAGS framework. Sparse dose regions were identified visually from the distribution of observed dose data displayed beneath the dose-response curves, and estimates in sparsely populated dose regions were interpreted cautiously. Bayesian models were fitted using Markov chain Monte Carlo methods with four chains, 40,000 iterations per chain, 20,000 burn-in iterations, and a thinning interval of 10. Model convergence was assessed by visual inspection of trace plots and by examining the potential scale reduction factor, also referred to as PSRF/R-hat, with values close to 1.00 considered to indicate adequate convergence.

### Risk of bias assessment

Two reviewers (S.L. and Y.T.) independently assessed the risk of bias of the included randomized controlled trials using the revised Cochrane risk-of-bias tool for randomized trials (RoB 2), with disagreements resolved through discussion or consultation with a third reviewer (L.Z.) when necessary [35]. The assessment focused on the effect of assignment to the intervention and covered five domains: bias arising from the randomization process, bias due to deviations from intended interventions, bias due to missing outcome data, bias in measurement of the outcome, and bias in selection of the reported result. Each domain was judged as low risk, some concerns, or high risk of bias according to the RoB 2 guidance. The overall risk-of-bias judgment for each study was classified as low risk when all domains were judged as low risk, as some concerns when at least one domain raised some concerns and no domain was judged as high risk, and as high risk when at least one domain was judged as high risk. The detailed RoB 2 assessments are presented in Additional file 1: Figs. S1 and S2.

### Certainty of evidence assessment

The certainty of evidence for the primary outcome (cfPWV) was assessed using the Grading of Recommendations Assessment, Development and Evaluation (GRADE) approach. Exercise-versus-control comparisons were graded primarily against the direct pairwise estimates, whereas exercise-versus-exercise comparisons were graded against the network estimates. Certainty was rated as high, moderate, low, or very low according to study design, risk of bias based on RoB 2, inconsistency or incoherence, indirectness, imprecision, and publication bias. Because this review included a network meta-analysis, certainty judgments were interpreted with particular attention to the amount of direct evidence, the precision of the estimates, and the consistency between direct and indirect evidence. Two reviewers independently performed the certainty assessment, and disagreements were resolved by discussion with a third reviewer. Detailed evidence profiles and reasons for downgrading are provided in Additional file 1: Table S18.

## Results

### Study selection

**Fig. 1:**
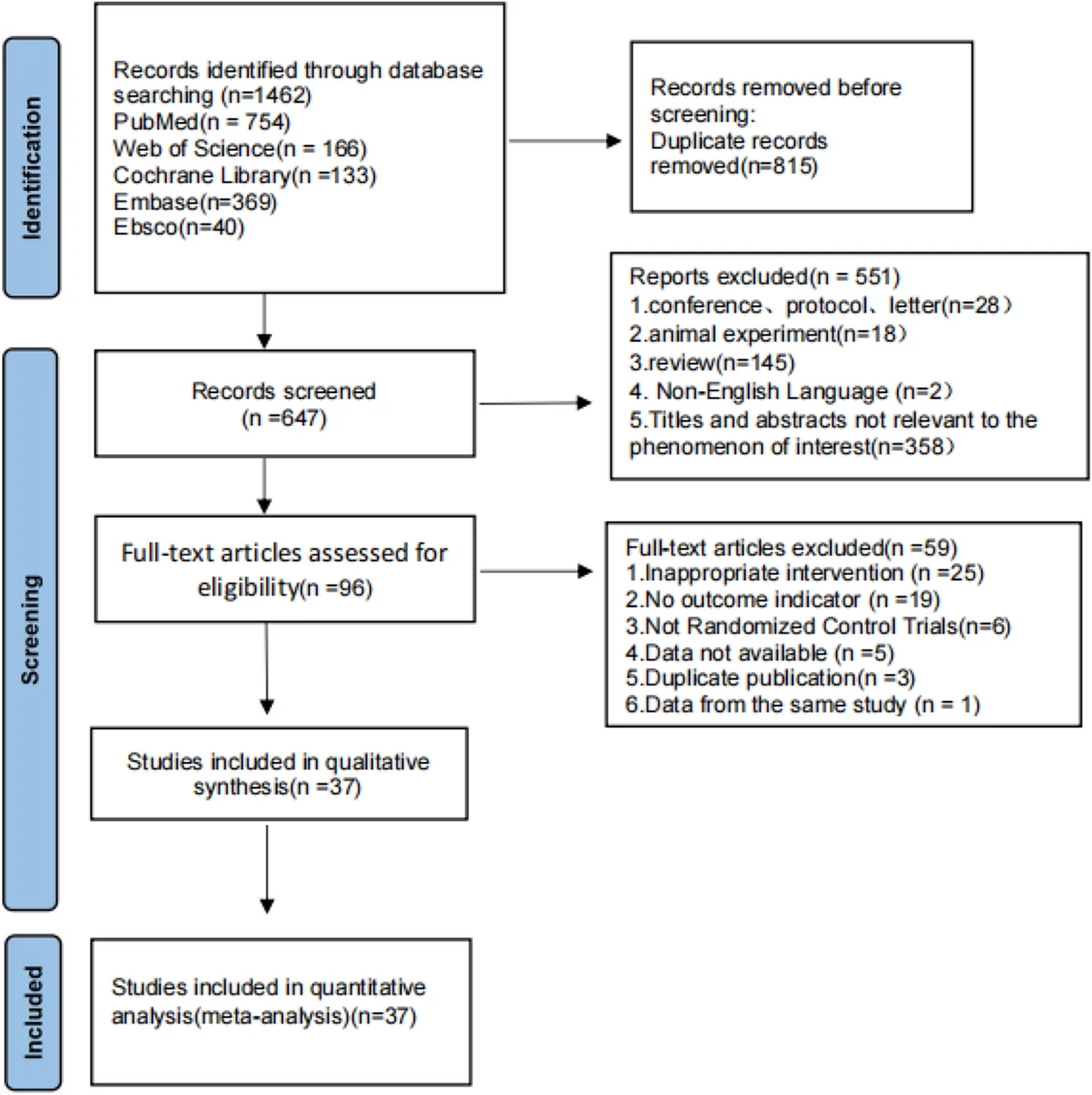
PRISMA flow diagram. Preferred Reporting Items for Systematic Reviews and Meta-Analyses (PRISMA) flow diagram of each stage of the review in risk-enriched adults

As shown in Fig. 1, a total of 1,462 records were identified through database searching, including 754 from PubMed, 166 from Web of Science, 133 from the Cochrane Library, 369 from Embase, and 40 from SPORTDiscus and CINAHL via EBSCOhost. After removal of 815 duplicate records, 647 records remained for title and abstract screening. A total of 551 reports were excluded at this stage. Ninety-six full-text articles were assessed for eligibility, of which 59 were excluded for predefined reasons, 37 studies were included in the qualitative synthesis and in the quantitative meta-analysis [36–72].

### Characteristics of the included studies

The characteristics of the included studies are summarized in Additional file 1: Table S6. A total of 37 randomized controlled trials involving 2,110 participants were included. The study populations comprised overweight or obese adults, adults with type 1 or type 2 diabetes, adults with hypertension or prehypertension, adults with metabolic syndrome, older adults, and postmenopausal women. Seven intervention categories were represented: AT, RT, CT, IT, WBVT, IHT, and ST. Across studies, participant age ranged from approximately 20 to 73 years and intervention duration ranged from 3 to 52 weeks. Overall, the included studies represented a heterogeneous but risk-enriched adult population. Although all populations were selected on the basis of elevated cardiovascular risk, they differed in population category, age, BMI, sex distribution, and underlying risk conditions, as summarized in Additional file 1: Table S6. Intervention characteristics also varied across exercise modalities, including intervention duration, session length, weekly frequency, and weekly exercise dose. These clinical and intervention-related characteristics were considered as potential effect modifiers when interpreting subgroup, heterogeneity, and network meta-analysis findings. Among the 37 included trials, the overall exercise-versus-control pairwise forest plot included 29 study-level exercise-versus-control estimates from 27 independent trials, with 975 participants in exercise arms and 697 participants in control arms as displayed in Fig. S3.

### Results of RoB 2 assessments

The RoB 2 assessment is summarized in Additional file 1: Figs. S1 and S2. Of the 37 included randomized controlled trials, 10 studies were judged as low risk of bias, 21 studies as having some concerns, and 6 studies as high risk of bias overall. Domain-level assessments showed that all studies were judged as low risk for measurement of the outcome, indicating that cfPWV assessment was generally unlikely to be a major source of bias. Some concerns were mainly related to the randomization process and selection of the reported result, whereas high-risk judgments were primarily driven by missing outcome data. Therefore, although most studies were not judged as high risk overall, only a minority were rated as low risk across all RoB 2 domains.

### Direct pairwise meta-analyses

#### Primary outcome

As shown in Additional file 1: Fig. S3, direct pairwise meta-analyses showed that exercise interventions were associated with lower cfPWV compared with control (WMD = -0.65, 95% CI: -1.13 to -0.18; I² = 94.4%). However, heterogeneity was considerable, indicating that this pooled estimate should be interpreted as an average effect across clinically and methodologically diverse trials rather than as a uniform expected effect. In subgroup analyses by exercise type, AT (WMD = -0.78, 95% CI: -1.37 to -0.19; I² = 87.6%) and WBVT (WMD = -0.40, 95% CI: -0.64 to -0.16; I² = 0.0%) were associated with lower cfPWV versus control. By contrast, RT (WMD = -0.27, 95% CI: -0.71 to 0.17; I² = 0.0%), CT (WMD = -0.21, 95% CI: -0.65 to 0.22; I² = 32.1%), IT (WMD = -1.45, 95% CI: -4.18 to 1.27; I² = 98.2%), and IHT (WMD = -0.97, 95% CI: -2.12 to 0.18; I² = 0.0%) did not reach statistical significance. The AT and IT comparisons showed considerable heterogeneity, and their pairwise estimates should therefore be interpreted cautiously. Sensitivity analyses using alternative correlation coefficients for imputing change-score SDs showed generally consistent directions across *r* = 0.25, *r* = 0.50, and *r* = 0.75. The overall exercise effect and aerobic training remained significant, whereas resistance training remained non-significant; however, combined training, whole-body vibration training, and isometric handgrip training showed sensitivity in statistical significance across assumptions (Additional file 1: Table S7). For IHT, statistical significance was observed only under the *r* = 0.75 assumption; because this comparison was informed by a single small trial, this should be interpreted as evidence instability rather than robust support for IHT efficacy. Visual inspection of the pairwise funnel plot did not show clear asymmetry (Additional file 1: Fig. S4).

#### Heterogeneity test

Leave-one-out sensitivity analyses were conducted for the AT versus control and IT versus control comparisons with considerable heterogeneity (Additional file 1: Table S8). For AT versus control, the pooled effect remained statistically significant after exclusion of any single study, and the largest reduction in heterogeneity was observed after excluding Kobayashi 2022 (I² = 43.9%, *P* = 0.066). This finding suggests that Kobayashi 2022 appeared influential in the AT subgroup and may have contributed to the observed heterogeneity, possibly in relation to its small three-arm design, postmenopausal normal-weight female sample, two aerobic-frequency arms against one control group, and relatively large cfPWV reductions after an 8-week intervention [37]. For IT versus control, which included only three studies in the direct pairwise forest plot, exclusion of Ghardashi-Afousi 2025 attenuated the pooled estimate and reduced heterogeneity (WMD = -0.29, 95% CI: -1.54 to 0.97; I² = 62.0%, *P* = 0.105). This suggests that this trial appeared influential in the IT subgroup and may have contributed to the observed heterogeneity, possibly reflecting its hypertensive older-adult sample and relatively large cfPWV reduction after a high-intensity interval protocol [60]. However, these leave-one-out findings should be considered exploratory and should not be interpreted as definitive evidence identifying the sources of heterogeneity. Overall, the AT result was directionally robust but heterogeneous, whereas the IT pairwise estimate should be interpreted cautiously because it was sensitive to a single trial.

To further explore the substantial heterogeneity observed in the overall exercise-versus-control analysis, subgroup analyses and meta-regression were performed (Table 1). A significant between-subgroup difference was observed for BMI category (P for subgroup difference = 0.018): the normal-weight subgroup showed a statistically significant reduction, the overweight subgroup showed a numerically large but imprecise reduction with a confidence interval crossing zero, and the obesity subgroup showed a smaller but statistically significant reduction. However, the corresponding meta-regression for BMI was not statistically significant (*P* = 0.196), so this pattern should be interpreted as exploratory rather than as evidence of a definitive linear BMI-effect relationship. Weekly exercise duration, age category, and intervention duration did not show significant subgroup differences or significant meta-regression results (all *P* > 0.05). Considerable residual heterogeneity remained in several subgroups, particularly for longer weekly exercise duration, younger participants, and interventions lasting at least 12 weeks. These analyses therefore only partly explained heterogeneity and should not be used to generate subgroup-specific prescription estimates.

**Table 1:** Subgroup analyses for overall exercise versus control

Group	Number of arms	WMD (95% CI)	I² (%)	*P* for heterogeneity	*P* for subgroup difference	*P* for Meta-regression
Week duration (min/week)						
< 150	15	-0.67 (-1.04,-0.29)	80.4	< 0.001	0.747	0.863
≥ 150	12	-0.85 (-1.91, 0.20)	96.2	< 0.001		
BMI(kg/m²)						
Normal weight	3	-1.08 (-1.68,-0.48)	87.5	< 0.001	0.018	0.196
Overweight	8	-1.05 (-2.62, 0.53)	92.5	< 0.001		
Obesity	18	-0.23 (-0.40,-0.06)	9.5	0.341		
Age(years)						
< 60	20	-0.60 (-1.27,0.06)	95.7	< 0.001	0.545	0.631
≥ 60	9	-0.87 (-1.43,-0.31)	76.2	< 0.001		
Duration (week)						
< 12 weeks	13	-0.60 (-1.01,-0.20)	85	< 0.001	0.917	0.944
≥ 12 weeks	16	-0.58 (-1.36,0.21)	95.9	< 0.001		

*cfPWV* carotid-femoral pulse wave velocity, *WMD* weighted mean difference

*P* for subgroup difference indicates the statistical test for differences between subgroups. *P* for meta-regression indicates the test for the linear association between the moderator and the effect size across study-level comparisons, and should be interpreted as exploratory because it was based on aggregate data. The number of arms varied across moderators because some studies lacked the relevant moderator data

### Results of network meta-analysis

**Fig. 2:**
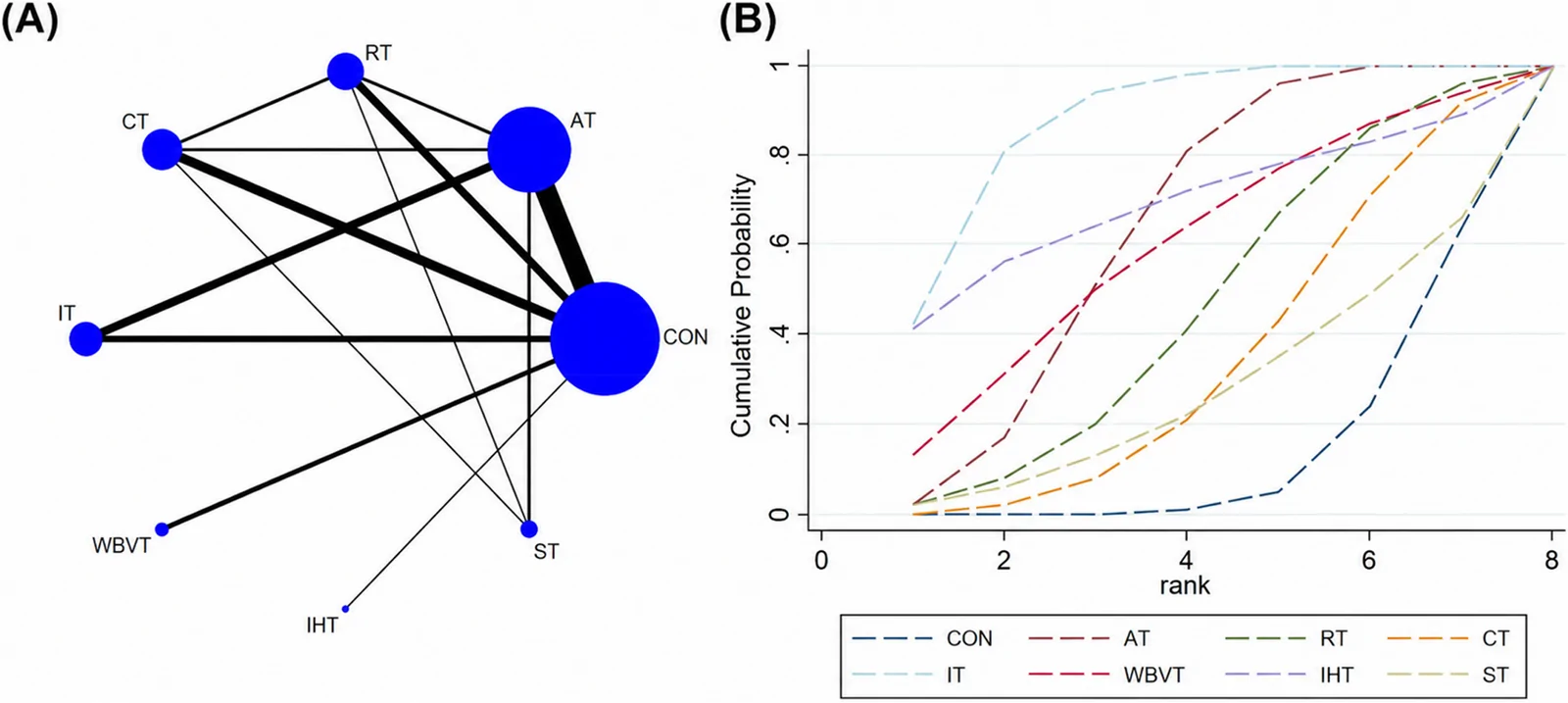
Network geometry and exploratory ranking probabilities. **(A)** Network plot of the included comparisons. The size of each node is proportional to the total number of participants assigned to each intervention, and the thickness of each line is proportional to the number of direct comparisons between two interventions. **(B)** Cumulative ranking probability plot of the included interventions. Curves closer to the upper left indicate higher ranking probabilities, but these rankings are exploratory and should be interpreted together with effect estimates, confidence intervals, direct evidence, network sparsity, and GRADE certainty. Abbreviations: CON, control; AT, aerobic training; RT, resistance training; CT, combined training; IT, interval training; WBVT, whole-body vibration training; IHT, isometric handgrip training; ST, stretching training; cfPWV, carotid-femoral pulse wave velocity

Figure 2A displays the NMA plot for eligible studies examining the effects of different exercise types on cfPWV. As shown in the plot, AT was the most frequently included intervention, whereas WBVT and IHT were less common.

Additional file 1: Fig. S5 illustrates the contributions of direct and indirect comparisons to the NMA, as well as the number of studies involved in each direct comparison. Loop-specific heterogeneity estimates, the global inconsistency model, and node-splitting analysis showed no statistically significant inconsistency in the network (Additional file 1: Tables S9-S11), supporting the use of the consistency model for the main analysis.

The results of the network meta-analysis are presented in Table 2. Compared with the control group, AT (WMD = -0.65, 95% CI: -1.08 to -0.22) and IT (WMD = -1.07, 95% CI: -1.66 to -0.49) were associated with lower cfPWV. RT (WMD = -0.41, 95% CI: -1.04 to 0.22), CT (WMD = -0.26, 95% CI: -0.82 to 0.31), WBVT (WMD = -0.64, 95% CI: -1.59 to 0.31), IHT (WMD = -0.97, 95% CI: -2.74 to 0.80), and ST (WMD = -0.11, 95% CI: -1.14 to 0.92) were not significantly different from control. Among active interventions, a very-low-certainty indirect network estimate suggested lower cfPWV with IT than CT (WMD = -0.82, 95% CI: -1.59 to -0.04), but no direct head-to-head evidence was available and this active-to-active comparison should not be interpreted as definitive superiority; other active comparisons were not statistically significant.

**Table 2:** League table for cfPWV network meta-analysis

AT							
-0.24 (-0.93,0.46)	RT						
-0.39 (-1.01,0.23)	-0.15 (-0.89,0.58)	CT					
0.42 (-0.15,1.00)	0.66 (-0.16,1.49)	**0.82 (0.04**,**1.59)**	IT				
-0.01 (-1.05,1.03)	0.23 (-0.91,1.37)	0.38 (-0.72,1.49)	-0.43 (-1.55,0.68)	WBVT			
0.32 (-1.50,2.14)	0.56 (-1.32,2.44)	0.71 (-1.14,2.57)	-0.10 (-1.97,1.76)	0.33 (-1.68,2.34)	IHT		
-0.54 (-1.52,0.45)	-0.30 (-1.41,0.81)	-0.15 (-1.22,0.93)	-0.96 (-2.08,0.16)	-0.53 (-1.93,0.88)	-0.86 (-2.91,1.19)	ST	
**-0.65 (-1.08**,**-0.22)**	-0.41 (-1.04,0.22)	-0.26 (-0.82,0.31)	**-1.07 (-1.66**,**-0.49)**	-0.64 (-1.59,0.31)	-0.97 (-2.74,0.80)	-0.11 (-1.14,0.92)	CON

Values are presented as WMDs with 95% CIs for cfPWV. Each cell represents the column-defining treatment minus the row-defining treatment. Negative WMD values favor the column-defining treatment, because lower cfPWV indicates greater improvement in central arterial stiffness; positive WMD values favor the row-defining treatment. Statistically significant comparisons are shown in bold when the 95% CI does not include 0

*AT* aerobic training, *CON* control, *CT* combined training, *IHT* isometric handgrip training, *IT* interval training, *RT* resistance training, *ST* stretching training, *WBVT* whole-body vibration training

Additional file 1: Fig. S6 displays the forest plots of eligible comparisons for the outcome indicator.

The SUCRA probability ranking results are presented as supportive summaries in Fig. 2B and Additional file 1: Table S13. Under the primary *r* = 0.50 assumption, IT showed the highest ranking probability (SUCRA = 87.7%), followed by IHT (69.0%) and AT (63.6%), whereas the control group had the lowest ranking probability (SUCRA = 13.5%). The apparently high ranking of IHT should be interpreted with particular caution because it was informed by a single small trial with wide uncertainty and should not be taken as evidence of genuine superiority. However, these rankings should be interpreted cautiously because several estimates were imprecise, some treatment nodes were supported by sparse evidence, and confidence intervals overlapped across several active comparisons. Therefore, SUCRA rankings were not used as the primary basis for judging comparative effectiveness or clinical superiority. Sensitivity analyses using *r* = 0.25 and *r* = 0.75 for imputing change-score SDs showed no material changes in the main network estimates: AT and IT remained associated with lower cfPWV than CON across assumptions (Additional file 1: Tables S12 and S13).

**Fig. 3:**
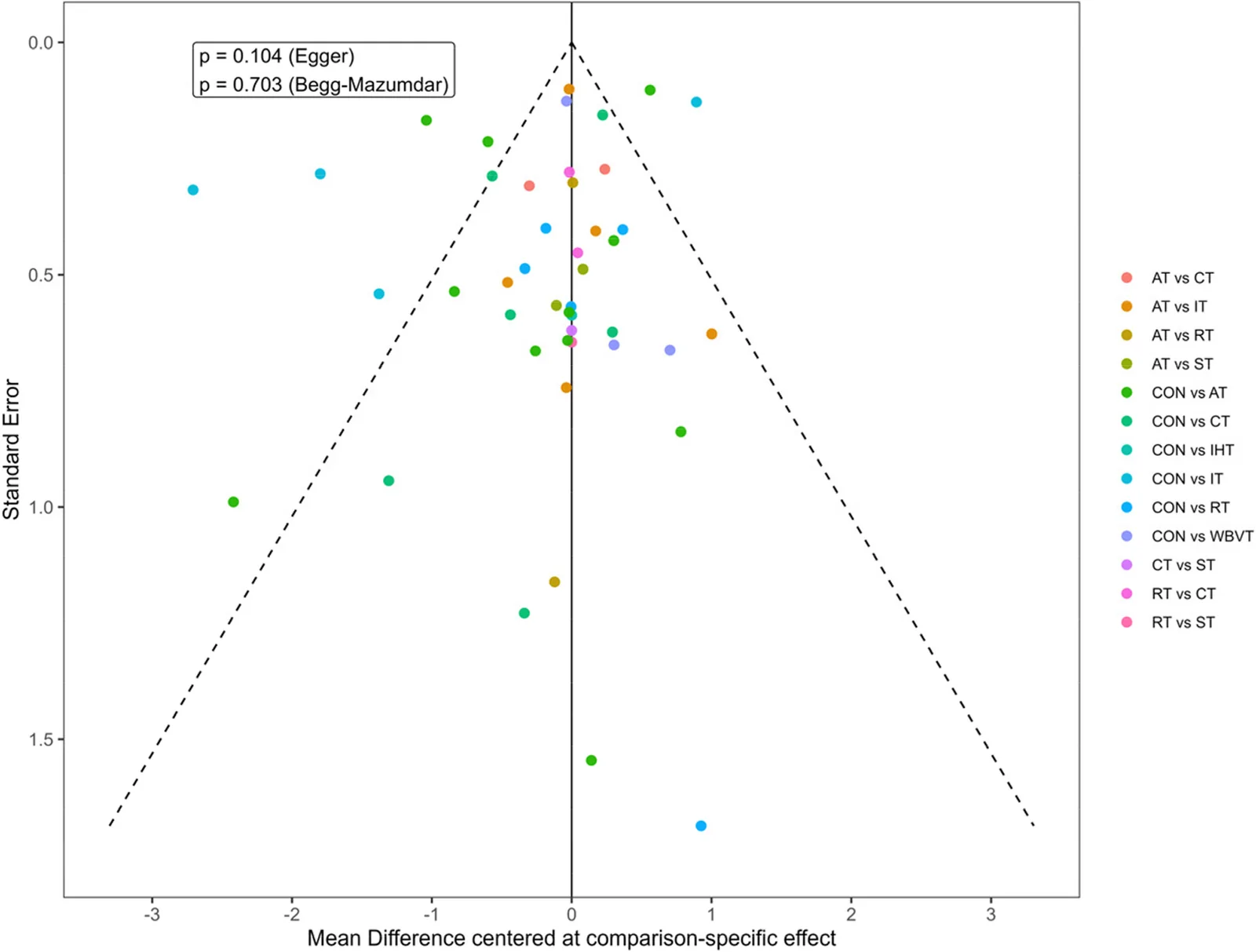
Comparison-adjusted funnel plot. Comparison-adjusted funnel plot of the network meta-analysis. CON, control; AT, aerobic training; RT, resistance training; CT, combined training; IT, interval training; WBVT, whole-body vibration training; IHT, isometric handgrip training; ST, stretching training

The comparison-adjusted funnel plot (Fig. 3) showed no obvious asymmetry on visual inspection, and the Egger and Begg-Mazumdar tests were not statistically significant (*P* = 0.104 and *P* = 0.703, respectively), suggesting no marked small-study effects in the network meta-analysis.

### Dose-response results

The key assumptions for network meta-analysis were assessed through evaluations of network connectivity, clinical and methodological comparability of potential effect modifiers, and statistical consistency. The distribution of potential effect modifiers across treatment nodes, including population category, age, sex distribution, BMI, baseline cfPWV, trial duration, and weekly exercise dose, is summarized in Additional file 1: Table S15; network connectivity and statistical consistency are presented in Additional file 1: Figs. S7-S9 and Table S14. For the dose-response analysis, different nonlinear models were compared using the Deviance Information Criterion (DIC). The quadratic random-effects model yielded the lowest DIC (87.06) and residual deviance (68.45), whereas the restricted cubic spline model (DIC = 231.35) and the Emax model (DIC = 340.51) showed poorer fit. Therefore, the quadratic model was retained as the final model for the dose-response analyses (Additional file 1: Table S16 and Additional file 1: Figs. S10-S13). Convergence diagnostics supported adequate model convergence, with trace plots showing stable and well-mixed chains without clear trends or separation, and PSRF/R-hat values close to 1.00 for the monitored parameters (Additional file 1: Table S17 and Figs. S14-S15).

**Fig. 4:**
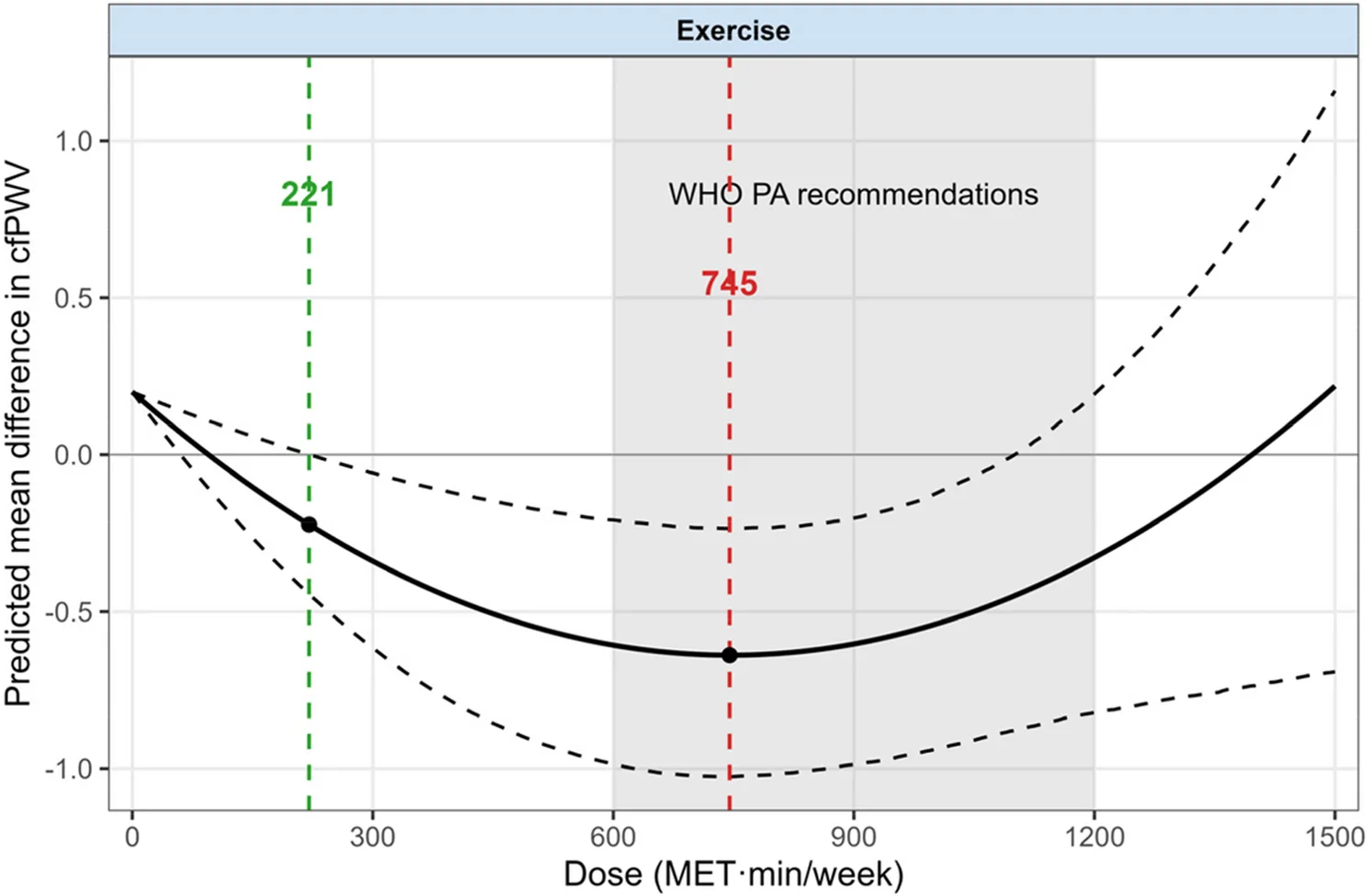
Overall dose-response relationship. Overall exercise dose-response relationship. The green line indicates the approximate model-derived lower crossing region at which the 95% credible interval first excluded 0, the red line indicates the dose region with the lowest fitted estimate, and the shaded area represents the WHO-recommended range of physical activity. Numeric labels in the figure are model-derived reference points and should be interpreted as approximate ranges rather than precise clinical thresholds

Figure 4 shows a nonlinear dose-response association between overall exercise dose and cfPWV. In this analysis, negative MD values indicated reductions in cfPWV, whereas positive values indicated no reduction or a less favorable direction of effect. In the primary dose categorization, the model-derived curve suggested that the lower crossing region at which the upper 95% credible interval first excluded 0 was approximately 200–300 MET·min/week, with the lowest fitted estimate occurring around 700–800 MET·min/week. These values describe the fitted model and should not be interpreted as a minimum effective dose or as precise clinical thresholds. The dose-response estimates should therefore be read as exploratory model-based ranges rather than as prescriptive dose targets. The fitted curve remained below zero across much of the WHO-recommended physical activity range, but uncertainty increased toward the upper end of the dose range where the observed evidence was thinner.

**Fig. 5:**
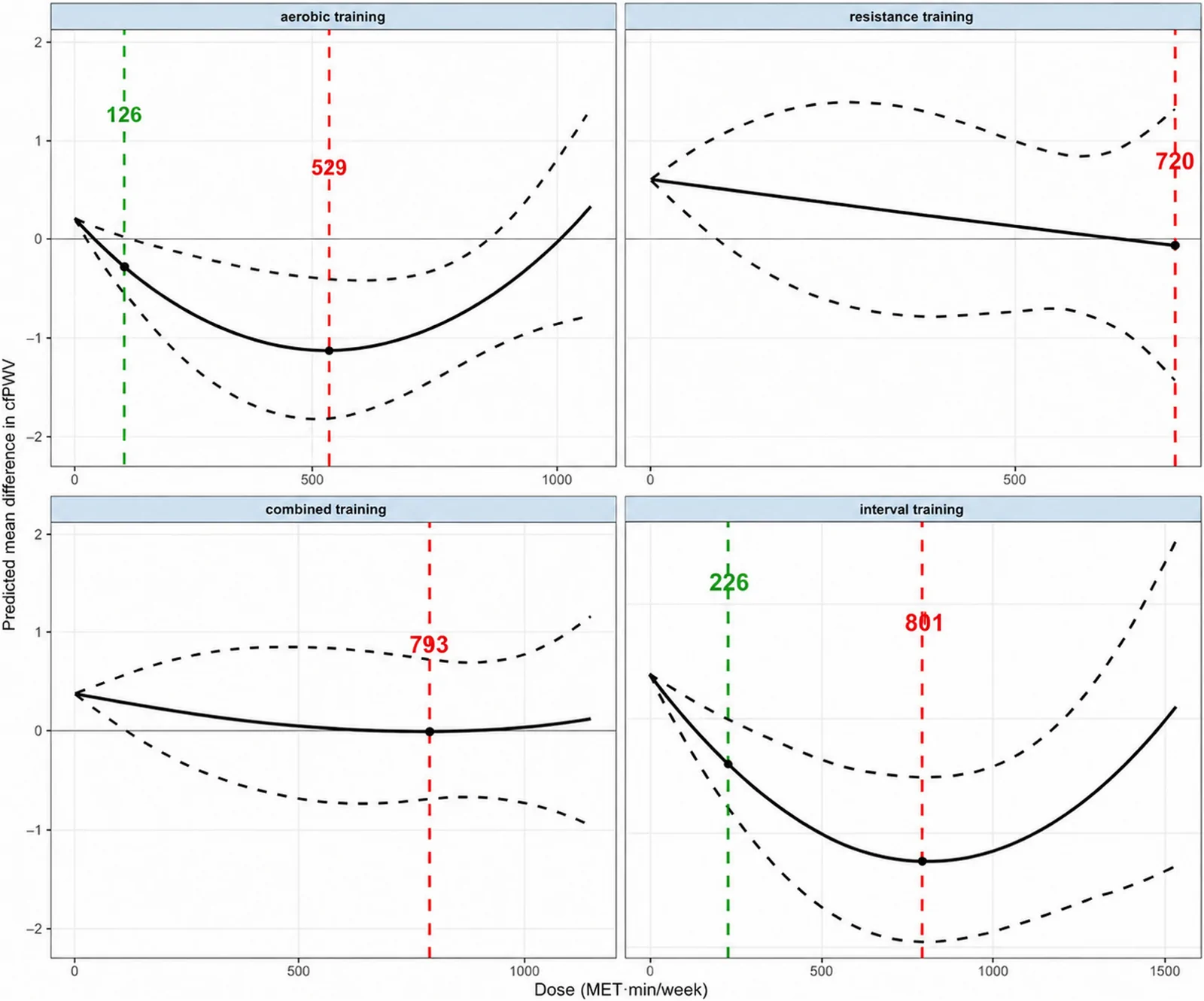
Exercise-type-specific dose-response relationships. Dose-response relationship by exercise type. The green line indicates the approximate lower dose region at which the 95% credible interval first excluded 0, and the red line indicates the dose region with the lowest fitted estimate for each displayed exercise type. Numeric labels in the figure are model-derived reference points and should be interpreted as approximate ranges rather than precise modality-specific thresholds

Figure 5 presents the dose-response relationships for different exercise types affecting cfPWV. The predicted results suggested the clearest and most interpretable nonlinear associations for aerobic training and interval training, for which MET·min/week more directly reflected the main exercise exposure. For aerobic training, the model-estimated lower crossing region appeared around 100–200 MET·min/week and the lowest fitted estimate around 500–600 MET·min/week. For interval training, the corresponding ranges were approximately 200–300 and 800–900 MET·min/week, respectively. These values should be interpreted as fitted-curve summaries and approximate ranges rather than modality-specific minimum effective doses or prescription thresholds. By contrast, resistance training and combined training showed relatively gradual and less certain dose-response trends, with wide uncertainty intervals and estimates close to or above zero in parts of the dose range. Whole-body vibration training and stretching training were shown only in supplementary dose-response plots with original data because of sparse evidence, and isometric handgrip training was not interpreted as a stable dose-response curve because only one direct study was available. Therefore, dose-response findings for modalities other than AT and IT should be regarded as exploratory rather than as definitive modality-specific dose prescriptions. Plots of the dose-response relationship including the original study dataset are shown in Additional file 1: Figs. S16 and S17.

Posterior ranking distributions for the dose-response model are provided in Additional file 1: Fig. S18 as exploratory supplementary information. These distributions were not used to identify a single best exercise dose or to establish dose-specific comparative advantage, because ranking probabilities in sparse dose regions can be unstable and should be interpreted together with the fitted dose-response curves, credible intervals, and the distribution of observed dose data.

### Certainty of evidence and study limitations

According to the GRADE approach, the certainty of evidence for cfPWV across the key comparisons ranged from moderate to very low. Moderate-certainty evidence was found for whole-body vibration training versus control and for aerobic training versus interval training, whereas the evidence for overall exercise, aerobic training, resistance training, combined training, interval training, isometric handgrip training, and several other comparisons was judged as low or very low. Downgrading was mainly due to study limitations identified by RoB 2, imprecision, sparse evidence, heterogeneity, and/or reliance on indirect estimates; inconsistency downgrading was applied where high pairwise heterogeneity was present, including overall exercise, AT, and IT versus control. Detailed certainty ratings and reasons for downgrading are presented in Additional file 1: Table S18.

The contribution plot of study limitations (Additional file 1: Figs. S19 and S20) showed that the network estimates were informed by a mixture of low-risk, some-concerns, and high-risk evidence, with low-risk and some-concerns evidence contributing comparable proportions and high-risk evidence accounting for a smaller but non-negligible share. At the study level, 10 of the 37 trials were judged as low risk across all RoB 2 domains, 21 studies raised some concerns, and 6 studies were judged as high risk overall. Taken together, these findings indicate that the comparative estimates should be interpreted with caution, especially for interventions supported by sparse evidence, wide uncertainty intervals, or a substantial contribution from studies with some concerns or high risk of bias. Accordingly, the ranking results should be viewed as supportive rather than definitive, and should not be interpreted in isolation from the corresponding effect estimates, their uncertainty, and the certainty-of-evidence ratings.

## Discussion

### Discussion of the pairwise meta-analysis

The present study focused specifically on heterogeneous risk-enriched adults with cardiovascular risk-related conditions or characteristics, including overweight or obesity, diabetes, hypertension or prehypertension, metabolic syndrome, older age, and postmenopausal status. In this broad, risk-enriched, and clinically heterogeneous population, the pairwise meta-analysis showed that exercise was associated with an average reduction in cfPWV overall. This finding supports the potential role of regular exercise in improving central arterial stiffness among adults with elevated cardiovascular risk; however, the considerable heterogeneity observed in the overall exercise-versus-control comparison indicates that the pooled effect should not be interpreted as a uniform expected response for every risk subgroup or exercise prescription. Among the modality-specific comparisons, AT and WBVT showed significant benefits versus control, whereas RT, CT, IT, and IHT did not reach statistical significance in the direct pairwise analyses. Taken together, these findings suggest that aerobic-based exercise currently has the most consistent direct pairwise support for reducing cfPWV in risk-enriched adults, while the evidence for other modalities appears more variable and may depend on the amount of available data, participant characteristics, intervention design, and between-study heterogeneity [73–75].

The significant result for AT is clinically plausible, because aerobic exercise is commonly prescribed across cardiometabolic and older-adult populations and has repeatedly been associated with lower PWV in adults with long-term conditions, in adults with CVD or high CVD risk, and in middle-aged and older populations [73–76]. Mechanistically, aerobic training-related reductions in arterial stiffness have been linked to improved nitric oxide bioavailability and endothelial function [77]. By contrast, the non-significant findings for RT, CT, IT, and IHT in pairwise analyses should not be interpreted as evidence of no effect. Rather, these results likely reflect a combination of limited study numbers in some subgroups, between-study heterogeneity, and substantial variation in exercise prescription [35, 78]. The significant WBVT result is noteworthy, but it should still be interpreted cautiously because this comparison was based on limited evidence from relatively specific populations, mainly postmenopausal women with elevated blood pressure [40, 48].

Considerable heterogeneity was observed in the overall exercise-versus-control analysis and remained evident in the AT and IT subgroup analyses. Subgroup analyses suggested that BMI category may partly explain heterogeneity; however, the BMI meta-regression was not statistically significant, and weekly exercise duration, age category, and intervention duration did not show significant explanatory effects. In the leave-one-out analysis, Kobayashi 2022 appeared influential in the AT subgroup, as its exclusion substantially reduced heterogeneity; this influence may be related to its small multi-arm aerobic-frequency design in postmenopausal normal-weight women with a relatively large short-term cfPWV response [37]. For IT, Ghardashi-Afousi 2025 appeared influential because its exclusion attenuated the pooled effect and reduced heterogeneity; this influence may reflect its hypertensive older-adult sample and relatively large response to HIIT [60]. These findings should be viewed as exploratory sensitivity results rather than definitive evidence identifying the causes of heterogeneity. They suggest that the AT conclusion was directionally robust but heterogeneous, whereas the IT estimate was more sensitive to a single high-response trial and should be interpreted cautiously.

### Discussion of the network meta-analysis

The network meta-analysis provided a broader comparative picture by combining direct and indirect evidence across seven exercise modalities. In contrast to the pairwise findings, the network estimates showed that AT and IT were associated with lower cfPWV than control, whereas RT, CT, WBVT, IHT, and ST were not. Conversely, the direct pairwise estimate for WBVT versus control was statistically significant, whereas the corresponding network estimate was not. This discrepancy may reflect the sparse and population-specific direct WBVT evidence, wider uncertainty after incorporating indirect evidence, and the limited ability of a small WBVT node to support robust comparative inference. In addition, a very-low-certainty indirect network estimate suggested lower cfPWV with IT than CT, but no direct head-to-head evidence was available and this active-to-active comparison should not be interpreted as definitive superiority; no other active intervention comparisons reached statistical significance. These effect-estimate results suggest that AT and IT currently have the clearest comparative support versus control in this cfPWV-specific network, but comparative differences among active exercise modalities remain uncertain and should not be inferred from ranking probabilities alone.

It should also be noted that the direct pairwise comparison for IT versus control did not reach statistical significance and showed very high heterogeneity, whereas the network estimate for IT versus control was statistically significant. This difference likely reflects the incorporation of indirect evidence and model-based synthesis in the network meta-analysis, together with the instability of the sparse and highly heterogeneous direct IT evidence. Therefore, the IT finding should be interpreted as low-certainty network evidence rather than as a robust direct-effect estimate.

SUCRA results were retained only as exploratory ranking probability summaries rather than as a primary basis for inference. Although IT showed a favorable ranking probability and this general pattern was similar across alternative correlation assumptions, several active-to-active estimates had overlapping confidence intervals, some comparisons were informed mainly by indirect or sparse direct evidence, and the IHT node was particularly imprecise. Therefore, clinical interpretation relies primarily on effect estimates, uncertainty intervals, direct evidence, network sparsity, and GRADE certainty rather than on SUCRA ranking alone [79, 80].

Another important point is that no significant inconsistency was detected in the network. This supports the use of the consistency model but should not be interpreted as establishing transitivity. Transitivity was considered broadly plausible only at the level of an overall prevention-oriented risk-enriched synthesis, but clinically fragile for disease-specific or active-to-active inference, because the included populations were clinically diverse and ranged from obesity and diabetes to hypertension, metabolic syndrome, older age, and postmenopausal status. These conditions and characteristics share elevated cardiovascular risk, but they may not respond identically to exercise. The descriptive summary of potential effect modifiers across treatment nodes (Additional file 1: Table S15) supports this cautious interpretation, showing that the included nodes differed in population composition, baseline cfPWV, age, BMI, sex distribution, trial duration, and weekly exercise dose. Thus, the network results are best interpreted as exploratory average comparative effects for heterogeneous risk-enriched adults rather than as uniform treatment effects across all clinical subgroups or definitive active-to-active comparative claims.

### Discussion of the dose-response analysis

As an additional exploratory component of the cfPWV-specific synthesis, this review examined exercise dose. However, the dose-response findings should be interpreted conservatively. The fitted model suggested a nonlinear association between overall exercise dose and cfPWV, with the lowest fitted estimate occurring around 700–800 MET·min/week. In the primary model, the lower crossing region occurred around 200–300 MET·min/week, but this value should be interpreted as an approximate model-derived range rather than an exact threshold because exercise protocols, population characteristics, MET assignment, dose categorization, and evidence density varied across studies. Taken together, these findings suggest a hypothesis-generating moderate-dose region with lower fitted cfPWV estimates for heterogeneous risk-enriched adults, whereas exact crossing points and apparent thresholds should not be interpreted as uniform exercise prescription recommendations.

At the exercise-type level, AT and IT showed the clearest and most interpretable nonlinear patterns. For AT, the model-estimated lower crossing region appeared around 100–200 MET·min/week and the lowest fitted estimate around 500–600 MET·min/week. For IT, the model-estimated lower crossing region appeared around 200–300 MET·min/week and the lowest fitted estimate around 800–900 MET·min/week. These values are fitted-curve summaries and approximate ranges, and should not be interpreted as definitive minimum effective doses. The exercise-type-specific dose-response findings should not be interpreted with the same level of confidence across all modalities. Dose quantification is more straightforward for aerobic- and interval-based exercise because the reported session duration and intensity are closely aligned with the main exercise exposure. By contrast, RT and CT showed gradual and less certain dose-response trends, and their estimated MET·min/week values may not fully capture resistance load, number of sets and repetitions, rest intervals, time under tension, or the relative contribution of aerobic and resistance components. Estimates near or above zero should also be interpreted as no clear cfPWV reduction rather than as a beneficial response. WBVT and ST remained unstable because of sparse data and wide credible intervals, and IHT could not support a stable dose-response interpretation because only one direct study was available. Therefore, the dose-response findings are most defensible for AT and IT, whereas the curves for RT, CT, WBVT, and ST should be regarded as exploratory and interpreted cautiously [81].

From a practical perspective, these dose-response findings indicate a cautious but potentially useful message for heterogeneous risk-enriched adults: the model suggested a possible moderate-dose region with lower fitted cfPWV estimates at the overall exercise level, with the most interpretable dose-response patterns observed for AT and IT. At the same time, these implications should not be overstated or interpreted as definitive prescription thresholds. The predefined dose categories should be understood as analytical dose nodes rather than biological thresholds or clinical cut-points, and the reported dose values should be read as approximate ranges rather than exact values. High-dose estimates should be interpreted carefully because direct evidence becomes thinner at the upper end of the dose range, increasing uncertainty around the curve [33]. Because the selected quadratic model imposes a parametric curve across the entire dose range, predictions toward sparsely populated dose regions, especially near the upper boundary, may be more strongly model-driven and should not be interpreted as directly observed dose effects. Accordingly, apparent differences between adjacent dose levels, especially in sparsely populated high-dose regions, should be interpreted as model-based predictions rather than precise clinical thresholds. In addition, dose estimation was likely more precise for aerobic- and interval-based exercise than for resistance-based, combined, whole-body vibration, isometric handgrip, or stretching interventions, for which total session duration may not fully reflect true active loading or physiological exposure [81]. Thus, the present findings are more helpful for identifying plausible dose regions with lower fitted cfPWV estimates, especially for AT and IT, than for prescribing a single optimal exercise dose across all exercise modalities or clinical subgroups. This interpretation reflects the use of MET·min/week as a pragmatic exposure-standardization metric rather than as a complete measure of modality-specific training load.

### Strengths and limitations

This study has several strengths. First, its main incremental contribution is the cfPWV-specific design, which avoids pooling different PWV measurement sites and preserves the clinical meaning of central arterial stiffness. Second, it focused on heterogeneous risk-enriched adults with cardiovascular risk-related conditions or characteristics rather than mixing healthy adults with high-risk populations, thereby improving the relevance of the findings for prevention-oriented interpretation and future exercise-prescription research. Third, by combining pairwise meta-analysis, frequentist network meta-analysis, GRADE certainty assessment, and exploratory Bayesian dose-response modeling, this study was able to summarize comparative exercise effects while providing additional hypothesis-generating dose information. Fourth, the review was based on a registered protocol, a structured search strategy, and explicit risk-of-bias and evidence-appraisal procedures.

Several limitations should also be acknowledged. First, considerable heterogeneity remained in the direct pairwise analyses, particularly for the overall comparison and the AT and IT subgroups. This limits confidence in the exact magnitude and generalizability of the pooled effects, even though the average direction generally favored exercise. Although subgroup, meta-regression, and sensitivity analyses were conducted where data allowed, residual heterogeneity may reflect incompletely reported factors such as baseline vascular status, medication use, adherence, supervision, intervention fidelity, and protocol-level differences. Second, the included risk-enriched populations were clinically heterogeneous, and differences in age, obesity severity, diabetic status, blood pressure status, menopausal status, baseline vascular condition, medication use, and responsiveness to exercise may have influenced the pooled estimates. Consequently, the findings should not be interpreted as subgroup-specific prescription estimates.

Third, the review was restricted to English-language publications, which may have introduced language bias and may have excluded relevant trials published in other languages. Publication-bias assessments should also be interpreted cautiously because the number of studies was limited for several modality-specific comparisons, reducing the power of funnel-plot inspection and Egger’s test. Fourth, the RoB 2 assessment showed that 10 of the 37 included studies were judged as low risk across all domains, whereas most studies raised some concerns and 6 were judged as high risk overall. This pattern indicates that study limitations should be considered when interpreting both the pairwise and network estimates. In addition, the GRADE assessment indicated that the certainty of evidence was generally low or very low across most comparisons, although selected comparisons reached moderate certainty, mainly because of differences in study limitations, imprecision, sparse evidence, heterogeneity, and indirectness where applicable (Additional file 1: Table S18).

Fifth, although sensitivity analyses using *r* = 0.25 and *r* = 0.75 were conducted for the pairwise and network analyses, the true within-participant pre-post correlations were unavailable for many trials; therefore, imputed change-score SDs may still have influenced the precision and weighting of some estimates. In addition, categorizing exact weekly exercise doses into predefined analytical dose nodes may have introduced exposure misclassification and may have smoothed local nonlinear dose-response patterns. This categorization should be considered a pragmatic modeling decision rather than a biologically discrete dose classification. Therefore, the estimated crossing regions and favorable dose ranges should be interpreted cautiously and should not be regarded as definitive clinical prescription thresholds.

Finally, although MET·min/week provided a common metric for comparing weekly exercise exposure across trials, dose estimation was more directly interpretable for AT and IT than for RT, CT, IHT, ST, and WBVT because total session duration may not fully reflect active loading time, resistance volume, rest intervals, or modality-specific physiological exposure. In particular, it did not capture resistance load, sets, repetitions, time under tension, vibration frequency or amplitude, or isometric contraction duty cycle. Accordingly, the dose-response curves for RT, CT, ST, and WBVT should be regarded as exploratory, IHT could not support a stable dose-response interpretation because only one direct study was available, and the dose-response interpretation is more robust for AT and IT. Overall, the dose-response estimates should be interpreted as model-derived and hypothesis-generating rather than clinically actionable thresholds. Further high-quality trials with standardized exercise prescriptions and more complete reporting are needed before specific dose thresholds can be recommended. Further trials in more clinically homogeneous subgroups and individual participant data meta-analyses are needed to clarify whether baseline disease status, sex, age, BMI, baseline cfPWV, and medication use modify exercise responsiveness.

## Conclusions

In heterogeneous risk-enriched adults with cardiovascular risk-related conditions or characteristics, low-certainty evidence suggests that exercise may be associated with an average improvement in cfPWV, but the pairwise evidence showed considerable between-study heterogeneity. Direct pairwise evidence suggested potential benefits for exercise overall, especially for aerobic training and whole-body vibration training; however, the WBVT finding was based on sparse direct evidence, and these findings should be interpreted as average effects across diverse populations and exercise prescriptions rather than as uniform expected effects. In the network meta-analysis, aerobic training and interval training were the only modalities associated with lower cfPWV than control. Ranking probabilities were retained only as exploratory supplementary summaries and were not used as the basis for clinical inference. Because the certainty of evidence was generally low or very low for most comparisons, and several comparisons were supported by sparse evidence, indirect estimates, or trials with study limitations, the interpretation prioritizes effect estimates, uncertainty intervals, direct evidence, and certainty-of-evidence ratings rather than SUCRA ranking probabilities. Dose-response analysis further suggested a nonlinear pattern, with the lowest fitted estimate occurring around 700–800 MET·min/week at the overall exercise level and an approximate lower crossing region around 200–300 MET·min/week. At the exercise-type level, aerobic training and interval training showed the clearest and most interpretable dose-response relationships, with the lowest fitted estimates around 500–600 and 800–900 MET·min/week, respectively. These dose ranges should be understood as model-derived, hypothesis-generating signals, not as definitive prescription thresholds for all exercise modalities or clinical subgroups. Dose-response findings for resistance training, combined training, stretching training, and whole-body vibration training should be interpreted more cautiously and regarded as exploratory because MET·min/week based on total session duration may not fully reflect active loading, contraction characteristics, vibration parameters, or modality-specific physiological exposure; isometric handgrip training could not support stable dose-response interpretation because only one direct study was available. Overall, these conclusions should be interpreted with caution because of clinical heterogeneity across included populations, heterogeneity across studies, sparse evidence for several modalities, uncertainty in dose estimation, study limitations identified by RoB 2, and the low or very low certainty of evidence for most comparisons.

## Supplementary Information

Additional file 1: Supplementary materials. DOCX. Search strategies, characteristics of included studies, RoB 2 assessments, sensitivity analyses, network inconsistency assessments, SUCRA rankings, dose-response model diagnostics, supplementary figures, and GRADE evidence profiles.

Supplementary Material 2.

## Data Availability

No datasets were generated or analysed during the current study.

## Abbreviations

AS: Arterial stiffness
AT: Aerobic training
cfPWV: Carotid-femoral pulse wave velocity
CI: Confidence interval
CON: Control
CrI: Credible interval
CT: Combined training
CVD: Cardiovascular disease
DIC: Deviance Information Criterion
GRADE: Grading of Recommendations Assessment, Development and Evaluation
IHT: Isometric handgrip training
IT: Interval training
MBNMA: Model-based network meta-analysis
MD: Mean difference
MET: Metabolic equivalent of task
NMA: Network meta-analysis
PRISMA: Preferred Reporting Items for Systematic Reviews and Meta-Analyses
PROSPERO: International Prospective Register of Systematic Reviews
PWV: Pulse wave velocity
RCT: Randomized controlled trial
RoB 2: Revised Cochrane risk-of-bias tool for randomized trials
RT: Resistance training
ST: Stretching training
SUCRA: Surface under the cumulative ranking curve
WBVT: Whole-body vibration training
WHO: World Health Organization
WMD: weighted mean difference
