# Supplementary material for: Exercise modalities and dose for improving arterial stiffness in risk-enriched adults: a systematic review and dose-response network meta-analysis
Source: BMC Cardiovasc Disord. 2026 Jul 9;26:864. doi: 10.1186/s12872-026-06208-7 (PMC13631993; doi:10.1186/s12872-026-06208-7)
Supplement: Supplementary file 1 [file 12872_2026_6208_MOESM1_ESM.docx]

**Additional file 1: Supplementary materials**

Exercise Modalities and Dose for Improving Arterial Stiffness in Risk-Enriched Adults: A Systematic Review and Dose-Response Network Meta-Analysis

Shiqi Liu^1^, Yanli Tan^1^, Liuhong Zang^1*^

^1^ Institute of Physical Education, Xinjiang Normal University, Urumqi, Xinjiang 830017, China

^*^Corresponding author: Liuhong Zang

E-mail address: zlh751225@126.com

Supplementary methods: Search strategies for PubMed, Embase, the Cochrane Library, Web of Science, and EBSCOhost, including SPORTDiscus and CINAHL, were developed separately for each database. All databases were searched from inception to 22 February 2026

**Table S1. Search strategy in PubMed(N=754)**

| **Step** | **Search strategy** | **Results** |
| --- | --- | --- |
| #1 | "Exercise"[MeSH Terms] OR "Exercise Therapy"[MeSH Terms] OR "exercis*"[Title/Abstract] OR "training"[Title/Abstract] OR "physical activity"[Title/Abstract] OR "aerobic"[Title/Abstract] OR "resistance"[Title/Abstract] OR "interval"[Title/Abstract] OR "high-intensity interval training"[Title/Abstract] OR "HIIT"[Title/Abstract] OR "walking"[Title/Abstract] OR "running"[Title/Abstract] OR "cycling"[Title/Abstract] OR "yoga"[Title/Abstract] OR "Tai Chi"[Title/Abstract] OR "pilates"[Title/Abstract] | 3389109 |
| #2 | "carotid-femoral pulse wave velocity"[Title/Abstract] OR "cfPWV"[Title/Abstract] OR "aortic pulse wave velocity"[Title/Abstract] OR "pulse wave velocity"[Title/Abstract] | 13656 |
| #3 | "obes*"[Title/Abstract] OR "overweight"[Title/Abstract] OR "hypertension"[Title/Abstract] OR "prehypertension"[Title/Abstract] OR "high blood pressure"[Title/Abstract] OR "blood pressure"[Title/Abstract] OR "diabetes"[Title/Abstract] OR "type 2 diabetes"[Title/Abstract] OR "type 1 diabetes"[Title/Abstract] OR "metabolic syndrome"[Title/Abstract] OR "older adults"[Title/Abstract] OR "elderly"[Title/Abstract] OR "aging"[Title/Abstract] OR "ageing"[Title/Abstract] OR "postmenopausal"[Title/Abstract] OR "sedentary"[Title/Abstract] OR "inactive"[Title/Abstract] | 2665362 |
| #4 | "randomized controlled trial"[Publication Type] OR "controlled clinical trial"[Publication Type] OR "random*"[Title/Abstract] OR "trial"[Title/Abstract] OR "controlled"[Title/Abstract] | 2939993 |
| #5 | #1 AND #2 AND #3 AND #4 | 754 |

**Table S2. Cochrane search results (N=133)**

| **Step** | **Search strategy** | **Results** |
| --- | --- | --- |
| #1 | exercis*:ti,ab,kw OR training:ti,ab,kw OR "physical activity":ti,ab,kw OR aerobic:ti,ab,kw OR resistance:ti,ab,kw OR interval:ti,ab,kw OR "high-intensity interval training":ti,ab,kw OR HIIT:ti,ab,kw OR walking:ti,ab,kw OR running:ti,ab,kw OR cycling:ti,ab,kw OR yoga:ti,ab,kw OR "Tai Chi":ti,ab,kw OR pilates:ti,ab,kw | 468970 |
| #2 | obes*:ti,ab,kw OR overweight:ti,ab,kw OR hypertension:ti,ab,kw OR prehypertension:ti,ab,kw OR "high blood pressure":ti,ab,kw OR diabetes:ti,ab,kw OR "type 2 diabetes":ti,ab,kw OR "type 1 diabetes":ti,ab,kw OR "metabolic syndrome":ti,ab,kw OR "older adults":ti,ab,kw OR elderly:ti,ab,kw OR aging:ti,ab,kw OR ageing:ti,ab,kw OR postmenopausal:ti,ab,kw OR sedentary:ti,ab,kw OR inactive:ti,ab,kw | 353713 |
| #3 | "carotid-femoral pulse wave velocity":ti,ab,kw OR cfPWV:ti,ab,kw | 694 |
| #4 | random*:ti,ab,kw OR trial:ti,ab,kw OR placebo:ti,ab,kw OR "controlled clinical trial":ti,ab,kw OR "randomized controlled trial":ti,ab,kw OR randomization:ti,ab,kw OR randomly:ti,ab,kw | 1710419 |
| #5 | #1 AND #2 AND #3 AND #4 | 133 |

**Table S3. Web of Science search results (N=166)**

| **Step** | **Search strategy** | **Results** |
| --- | --- | --- |
| #1 | exercis* OR training OR "physical activity" OR aerobic OR resistance OR interval OR "high intensity interval training" OR HIIT OR walking OR running OR cycling OR yoga OR "Tai Chi" OR pilates | 9510028 |
| #2 | "carotid-femoral pulse wave velocity" OR "carotid femoral pulse wave velocity" OR cfPWV | 2779 |
| #3 | obes* OR overweight OR hypertension OR prehypertension OR "high blood pressure" OR diabetes OR "type 2 diabetes" OR "type 1 diabetes" OR "metabolic syndrome" OR "older adults" OR elderly OR aging OR ageing OR postmenopausal OR sedentary OR inactive | 7251166 |
| #4 | random* OR trial OR placebo OR randomly OR "controlled clinical trial" OR "randomized controlled trial" | 4469331 |
| #5 | #1 AND #2 AND #3 AND #4 | 166 |

**Table S4. EBSCOhost search results for SPORTDiscus and CINAHL (N=40)**

| **Step** | **Search strategy** | **Results** |
| --- | --- | --- |
| #1 | TI (exercis* OR training OR "physical activity" OR aerobic OR resistance OR interval OR "high intensity interval training" OR HIIT OR walking OR running OR cycling OR yoga OR "Tai Chi" OR pilates) OR AB (exercis* OR training OR "physical activity" OR aerobic OR resistance OR interval OR "high intensity interval training" OR HIIT OR walking OR running OR cycling OR yoga OR "Tai Chi" OR pilates) | 2676240 |
| #2 | TI ("carotid femoral pulse wave velocity" OR "carotid-femoral pulse wave velocity" OR cfPWV) OR AB ("carotid femoral pulse wave velocity" OR "carotid-femoral pulse wave velocity" OR cfPWV) | 1008 |
| #3 | TI (obes* OR overweight OR hypertension OR prehypertension OR "high blood pressure" OR diabetes OR "type 2 diabetes" OR "type 1 diabetes" OR "metabolic syndrome" OR "older adults" OR elderly OR aging OR ageing OR postmenopausal OR sedentary OR inactive) OR AB (obes* OR overweight OR hypertension OR prehypertension OR "high blood pressure" OR diabetes OR "type 2 diabetes" OR "type 1 diabetes" OR "metabolic syndrome" OR "older adults" OR elderly OR aging OR ageing OR postmenopausal OR sedentary OR inactive) | 1310190 |
| #4 | TI (random* OR trial OR placebo OR randomly OR "controlled clinical trial" OR "randomized controlled trial") OR AB (random* OR trial OR placebo OR randomly OR "controlled clinical trial" OR "randomized controlled trial") | 1746967 |
| #5 | #1 AND #2 AND #3 AND #4 | 40 |

**Table S5. Embase search results (N=369)**

| **Step** | **Search strategy** | **Results** |
| --- | --- | --- |
| #1 | 'exercise'/exp OR 'exercise therapy'/exp OR exercis*:ti,ab OR 'exercise training':ti,ab OR 'aerobic training':ti,ab OR 'resistance training':ti,ab OR 'interval training':ti,ab OR 'high-intensity interval training':ti,ab OR HIIT:ti,ab OR walking:ti,ab OR running:ti,ab OR cycling:ti,ab OR 'isometric handgrip':ti,ab OR 'whole-body vibration':ti,ab OR stretching:ti,ab | 1170760 |
| #2 | 'carotid-femoral pulse wave velocity':ti,ab OR 'carotid femoral pulse wave velocity':ti,ab OR cfPWV:ti,ab OR 'aortic pulse wave velocity':ti,ab OR 'aortic PWV':ti,ab | 8937 |
| #3 | obes*:ti,ab OR overweight:ti,ab OR hypertension:ti,ab OR prehypertension:ti,ab OR diabetes:ti,ab OR 'type 2 diabetes':ti,ab OR 'type 1 diabetes':ti,ab OR 'metabolic syndrome':ti,ab OR 'older adults':ti,ab OR elderly:ti,ab OR aging:ti,ab OR ageing:ti,ab OR postmenopausal:ti,ab OR sedentary:ti,ab OR inactive:ti,ab | 3609689 |
| #4 | 'randomized controlled trial'/exp OR 'controlled clinical trial'/exp OR random*:ti,ab OR trial:ti,ab OR randomly:ti,ab OR placebo:ti,ab | 3748260 |
| #5 | #1 AND #2 AND #3 AND #4 | 369 |

## Table S6. Characteristics of the included studies.

| **Author and published year** | **Characteristics of subject** | | | | **Interventions information** | | | | |
| --- | --- | --- | --- | --- | --- | --- | --- | --- | --- |
|  | **Sample size** | **age** | **BMI** | **Population category** | **Means of intervention** | **Exercise duration** | **Period and frequency** | **Exact dose** | **Rounded dose used in dose-response model** |
|  | **(%males)** | **(mean [SD])** | **(mean [SD])** |  |  | **(minutes)** | **(week × [times/week])** | **(MET·min/week)** | **(MET·min/week)** |
| Heydari 2013 | 20(100) | 24.9(4.3) | 28.4(2.4) | Overweight | IT | 20 | 12*3 | 480 | 600 |
|  | 18(100) |  | 29(3.9) |  | CON |  |  | 0 | 0 |
| Kobayashi 2022 | 15(0) | 62.5(2.8) | 22.4(1.6) | Postmenopausal Women | AT | 30 | 8*2 | 270 | 300 |
|  | 15(0) | 62.5(2.8) | 22.8(1.1) |  | AT | 30 | 8*4 | 540 | 600 |
|  | 15(0) | 60.9(3.9) | 22.7(1.4) |  | CON |  |  | 0 | 0 |
| Cortez-Cooper 2008 | 13(23.1) | 52(7.2) | 26.8(4.0) | Overweight | RT | 40 | 13*3 | 367.5 | 300 |
|  | 12(25) | 51(3.5) | 27.0(3.8) |  | CT | 40 | 13*4 | 900 | 900 |
|  | 12(50) | 54(6.9) | 25.7(3.8) |  | ST | 40 | 13*4 | 241.5 | 300 |
| Slivovskaja 2018 | 84(40.5) | 53.89(6.43) | 30.89(3.97) | Metabolic syndrome | AT | 35 | 8*5 | 875 | 900 |
|  | 42(57.1) | 52.01(7.67) | 30.97(3.51) |  | CON |  |  | 0 | 0 |
| Figueroa 2015 | 24(NA) | 57(3.54) | 34.2(4.2) | Postmenopausal Women | WBVT | 20 | 12*3 | 225 | 300 |
|  | 12(NA) | 58(3.46) | 32.2(7.27) |  | CON |  |  | 0 | 0 |
| Lin 2021 | 13(46.2) | 66(7.21) | 23.8(3.97) | Older persons | AT | 30 | 8*3 | 405 | 300 |
|  | 12(50) | 69(7.62) | 26.5(5.54) |  | CT | 30 | 8*3 | 270 | 300 |
|  | 9(44.4) | 70(9) | 25.4(3.60) |  | CON |  |  | 0 | 0 |
| Farah 2018 | 32(30) | 60.12(8) | 30.58(5.62) | Hypertension | IHT | 12 | 12*3 | 124 | 300 |
|  | 16(31) | 58(12) | 31.7(5.2) |  | CON |  |  | 0 | 0 |
| Guimarães 2010 | 16(43.8) | 50(8) | 28(4) | Hypertension | CT | 60 | 16*3 | 1020 | 900 |
|  | 16(25) | 45(9) | 29(5) |  | CT | 60 | 16*3 | 1020 | 900 |
|  | 11(18.2) | 47(6) | 26(5) |  | CON |  |  | 0 | 0 |
| Croymans 2014 | 28(100) | 21.5(2.22) | 31.1(2.22) | Obesity | RT | 60 | 12*3 | 720 | 600 |
|  | 8(100) | 21.87(1.48) | 33.17(2.59) |  | CON |  |  | 0 | 0 |
| DeVallance 2016 | 13(NA) | 51(10.8) | 32(3.6) | Metabolic syndrome | RT | 45 | 8*3 | 472.5 | 600 |
|  | 16(NA) | 51(16) | 33(8.0) |  | CON |  |  | 0 | 0 |
| Gómez-Sánchez 2022 | 127(33.1) | 47.96(9.95) | 32.71(3.27) | Obesity | AT | NA | 12*NA | NA | NA |
|  | 126(27.8) | 47.78(9.62) | 33.01(3.31) |  | CON |  |  | 0 | 0 |
| Cox 2024 | 23(60.9) | 59(8.8) | 32.6(5.1) | Type 2 diabetes | CT | 26 | 8*3 | 372 | 300 |
|  | 23(60.9) | 60.1(7.3) | 34(6.6) |  | CT | 52.5 | 8*4 | 1095 | 1200 |
|  | 23(60.9) | 59.4(10.2) | 33.9(6.4) |  | CON |  |  | 0 | 0 |
| Figueroa 2014 | 13(0) | 55.5(2.5) | 33.6(4.7) | Hypertension | WBVT | NA | 12*3 | NA | NA |
|  | 12(0) | 56.4(3.5) | 36(3.1) |  | CON |  |  | 0 | 0 |
| Chang 2025 | 21(50) | 62.9(7.63) | 30.05(5.58) | Type 2 diabetes | AT | 22 | 8*7 | 616 | 600 |
|  | 14(52) | 63.2(8.5) | 29.6(4.1) |  | CON |  |  | 0 | 0 |
| Banks 2024 | 13(61.5) | 52(6) | 29.9(4.2) | Hypertension | RT | 40 | 9*3 | 420 | 300 |
|  | 13(61.5) | 55(6) | 28(4.3) |  | CON |  |  | 0 | 0 |
| Blumenthal 2021 | 90(52) | 62(9) | 36(6) | Hypertension | AT | 45 | 16*3 | 720 | 600 |
|  | 50(52) | 63(9) | 36(5) |  | CON |  |  | 0 | 0 |
| Choo 2014 | 50(0) | 42.2(9.5) | 28.5(0.54) | obesity | AT | 60 | 36*3 | 810 | 900 |
|  | 30(0) | 46(10) | 27.9(0.58) |  | RT | 60 | 36*3 | 630 | 600 |
|  | 30(0) | 41.8(6.6) | 29.1(0.79) |  | CT | 60 | 36*3 | 945 | 900 |
| Donley 2014 | 11(27) | 46(13.27) | 38(6.63) | Metabolic syndrome | AT | 60 | 8*3 | 1080 | 1200 |
|  | 11(45) | 44(9.95) | 34(6.63) |  | CON |  |  | 0 | 0 |
| Fernandez-del-Valle 2018 | 6(0) | 22.69(3.35) | 34.19(3.03) | obesity | RT | 50 | 3*3 | 600 | 600 |
|  | 5(0) | 25(4.47) | 34.05(3.68) |  | CON |  |  | 0 | 0 |
| Jamka 2021 | 44(0) | 55(7) | 35.45(4.51) | obesity | AT | 60 | 12*3 | 810 | 900 |
|  | 41(0) | 55(7) | 35(4.32) |  | CT | 60 | 12*3 | 615 | 600 |
| Reed 2024 | 18(32) | 53(7) | 30.2(4.5) | Hypertension | AT | 35 | 12*3 | 630 | 600 |
|  | 8(33) | 51(7) | 30.2(4.6) |  | CON |  |  | 0 | 0 |
| Scott 2019 | 5(71.4) | 29(7.9) | 29.2(3.2) | Type 1 diabetes | IT | 19 | 6*3 | 408 | 300 |
|  | 6(71.4) | 29(13.2) | 25.3(3.2) |  | AT | 40 | 6*3 | 540 | 600 |
| Madden 2013 | 25(52) | 68.5(4.5) | 30.9(5) | Older persons | AT | 60 | 24*3 | 1080 | 1200 |
|  | 27(63) | 70(4.2) | 28.6(4.2) |  | CON |  |  | 0 | 0 |
| Stewart 2005 | 40(49) | 63(5.46) | 29.4(3.83) | Hypertension | CT | NA | 26*3 | 1147.5 | 1200 |
|  | 42(49) | 64.1(6.31) | 29.7(5) |  | CON |  |  | 0 | 0 |
| Ghardashi-Afousi 2025 | 40(55) | 60.15(5.95) | 27.28(1.36) | Hypertension | IT | 55 | 12*3 | 936 | 900 |
|  | 40(60) | 59.47(6.38) | 27.04(1.36) |  | CON |  |  | 0 | 0 |
| Okamoto 2018 | 22(50) | 72(4.7) | 22.2(2.3) | Older persons | IT | 30 | 20*3 | 585 | 600 |
|  | 24(41.6) | 73(4.9) | 22.2(2.0) |  | AT | NA | 20*3 | NA | NA |
| Fujie 2022 | 17(41.2) | 65.5(8.2) | 24.41(5.36) | Older persons | AT | 55 | 8*3 | 990 | 900 |
|  | 16(37.5) | 67.4(6) | 21.19(4.36) |  | CON |  |  | 0 | 0 |
| Tomoto 2023 | 28(25) | 68.2(5.3) | 25.8(3.9) | Older persons | AT | 40 | 52*4 | 1080 | 1200 |
|  | 27(30) | 67.8(4.9) | 26.8(3.6) |  | ST | 40 | 52*4 | 316 | 300 |
| Magalhães 2019 | 16(44) | 59.7(6.5) | 31.1(5) | Type 2 diabetes | CT | 42 | 52*3 | 744.3 | 600 |
|  | 13(70) | 56.7(8.3) | 30.1(5.7) |  | CT | 34 | 52*3 | 744.3 | 600 |
|  | 22(50) | 59(8.1) | 30.7(5) |  | CON |  |  | 0 | 0 |
| de Oliveira 2020 | 11(0) | 29.4(4.6) | 34.1(3.6) | Obesity | IT | NA | 8*NA | 790.5 | 900 |
|  | 14(0) | 27(5) | 35.7(2.4) |  | AT | 41 | 8*3 | 553.5 | 600 |
| Shiotsu 2018 | 30(100) | 70(4.3) | 23.85(2.61) | Older persons | CT | 40 | 10*2 | 480 | 600 |
|  | 10(100) | 71(4.4) | 23.8(2.5) |  | CON |  |  | 0 | 0 |
| Tomoto 2021 | 18(45) | 64.8(6.4) | 27.3(4.8) | Older persons | AT | 30 | 52*4 | 1080 | 1200 |
|  | 28(47) | 66.1(6.8) | 28.2(4.2) |  | ST | 30 | 52*4 | 310 | 300 |
| Way 2020 | 12(50) | 54.8(2.4) | 34.3(3.7) | Type 2 diabetes | AT | 55 | 12*3 | 742.5 | 600 |
|  | 12(58) | 56.9(2.1) | 37.5(5.4) |  | IT | 19 | 12*3 | 484.5 | 600 |
|  | 11(64) | 51.9(1.4) | 35.8(5.7) |  | CON |  |  | 0 | 0 |
| Alvarez-Alvarado 2017 | 25(0) | 20(5) | 30.7(3.5) | Obesity | WBVT | 20 | 6*3 | 154 | 300 |
|  | 13(0) | 21(3.6) | 31.3(4) |  | CON |  |  | 0 | 0 |
| Petrick 2021 | 11(100) | 35.3(15.1) | 33.9(2.4) | Obesity | AT | 35 | 6*5 | 787.5 | 900 |
|  | 12(100) | 39.4(14.9) | 34.1(4.3) |  | IT | 15 | 6*3 | 405 | 300 |
| Dobrosielski 2012 | 70(59) | 57(6) | 33(0.6) | Type 2 diabetes | CT | 60 | 24*3 | 607.5 | 600 |
|  | 70(57) | 56(6) | 33.6(0.5) |  | CON |  |  | 0 | 0 |
| Deiseroth 2019 | 42(47.4) | 58(5) | 33.3(3) | Obesity | IT | 60 | 12*3 | 1530 | 1500 |
|  | 37(63.3) | 57(6) | 33.1(5.1) |  | CON |  |  | 0 | 0 |

Note: CON, control; AT, aerobic training; RT, resistance training; CT, combined training; IT, interval training; WBVT, whole-body vibration training; IHT, isometric handgrip training; ST, stretching training. NA: not available. Rounded dose refers to the nearest predefined dose category used in the dose-response model (0, 300, 600, 900, 1200 and 1500 MET·min/week).

## Fig. S1. RoB 2 assessment of included randomized controlled trials.

## Fig. S2. Summary plot of RoB 2 judgments across the five risk-of-bias domains and overall risk of bias.

**Fig. S3. Forest plot of the effects of exercise versus control on carotid-femoral pulse wave velocity (cfPWV) in risk-enriched adults.**

**Table S7. Sensitivity analyses using alternative correlation coefficients for imputing change-score SDs**

| **Comparison** | **r = 0.25**  **WMD (95% CI)** | **I² (%)** | **r = 0.50**  **WMD (95% CI)** | **I² (%)** | **r = 0.75**  **WMD (95% CI)** | **I² (%)** |
| --- | --- | --- | --- | --- | --- | --- |
| Overall exercise vs CON | -0.64 (-0.99, -0.29) | 78.3 | -0.65 (-1.13, -0.18) | 94.4 | -0.63 (-0.97, -0.30) | 93.1 |
| AT vs CON | -0.75 (-1.33, -0.17) | 75.7 | -0.78 (-1.37, -0.19) | 87.6 | -0.79 (-1.36, -0.22) | 92.9 |
| RT vs CON | -0.26 (-0.72, 0.20) | 0.0 | -0.27 (-0.71, 0.17) | 0.0 | -0.24 (-0.54, 0.05) | 0.0 |
| CT vs CON | -0.52 (-0.73, -0.32) | 0.0 | -0.21 (-0.65, 0.22) | 32.1 | -0.15 (-0.62, 0.32) | 60.0 |
| IT vs CON | -1.35 (-3.42, 0.71) | 94.4 | -1.45 (-4.18, 1.27) | 98.2 | -1.34 (-3.37, 0.70) | 98.1 |
| WBVT vs CON | -0.39 (-0.81, 0.04) | 0.0 | -0.40 (-0.64, -0.16) | 0.0 | -0.52 (-0.96, -0.07) | 30.0 |
| IHT vs CON | -0.97 (-2.37, 0.43) | 0.0 | -0.97 (-2.12, 0.18) | 0.0 | -0.97 (-1.80, -0.14) | 0.0 |

Notes: Values are WMDs with 95% CIs from random-effects pairwise meta-analyses. Negative WMD values favor exercise because lower cfPWV indicates improvement. r denotes the assumed pre–post correlation coefficient used to impute change-score SDs. r = 0.50 was used as the primary assumption; r = 0.25 and r = 0.75 were used for sensitivity analyses.AT, aerobic training; CI, confidence interval; CON, control; CT, combined training; IHT, isometric handgrip training; IT, interval training; RT, resistance training; SD, standard deviation; WBVT, whole-body vibration training; WMD, weighted mean difference.

**Fig. S4. Funnel plot of carotid–femoral pulse wave velocity.**

**Table S8. Leave-one-out sensitivity analysis for carotid-femoral pulse wave velocity**

| **Intervention** | **Excluded literature** | **WMD (95% CI)** | **I^2^** | **P for heterogeneity** |
| --- | --- | --- | --- | --- |
| **AT vs CON** | **Kobayashi 2022** | **-0.52 (-0.92,-0.13)** | **43.9** | **0.066** |
|  | Slivovskaja 2018 | -0.85 (-1.53,-0.17) | 87.3 | p<0.001 |
|  | Lin 2021(a) | -0.77 (-1.38,-0.17) | 88.8 | p<0.001 |
|  | Gómez-Sánchez 2022 | -0.92 (-1.42,-0.41) | 70.3 | p<0.001 |
|  | Chang 2025 | -0.71(-1.33,-0.09) | 88.7 | p<0.001 |
|  | Blumenthal 2021 | -0.85 (-1.48,-0.22) | 88.4 | p<0.001 |
|  | Donley 2014 | -0.79 (-1.42, -0.16) | 88.8 | p<0.001 |
|  | Reed 2024 | -0.77 (-1.40, -0.14) | 88.8 | p<0.001 |
|  | Madden 2013 | -0.77 (-1.39,-0.15) | 88.8 | p<0.001 |
|  | Fujie 2022 | -0.73 (-1.37,-0.08) | 88.7 | p<0.001 |
|  | Way 2020(a) | -0.77 (-1.40, -0.14) | 88.8 | p<0.001 |
| **IT vs CON** | **Ghardashi-Afousi 2025** | **-0.29 (-1.54,0.97)** | **62** | **0.105** |
|  | Way 2020(b) | -1.60 (-5.10, 1.90) | 99.1 | p<0.001 |
|  | Deiseroth 2019 | -2.37 (-4.56,-0.19) | 88.3 | 0.004 |

**Fig. S5. Contributions of direct and indirect comparisons to NMA and the number of studies of each direct comparison.**

Note: A: Control; B: Aerobic training; C: Resistance training; D: Combined training; E:interval training; F:WBVT; G:Isometric handgrip training; H:Stretching training.

Inconsistency of outcome indicator tested by loop-specific heterogeneity estimates, inconsistency model and node splitting analysis.

Note: A: Control; B: Aerobic training; C: Resistance training; D: Combined training; E:interval training; F:WBVT; G:Isometric handgrip training; H:Stretching training.

**Table S9. Loop-specific heterogeneity estimates.**

| **Loop** | **IF** | **seIF** | **z_value** | **p_value** | **CI_95** | **Loop_Heterog_tau2** |
| --- | --- | --- | --- | --- | --- | --- |
| CON-AT-RT | 0.564 | 0.885 | 0.637 | 0.524 | (0.00,2.30) | 0.594 |
| CON-AT-CT | 0.424 | 0.731 | 0.58 | 0.562 | (0.00,1.86) | 0.536 |
| CON-AT-IT | 0.522 | 0.945 | 0.553 | 0.58 | (0.00,2.37) | 1.406 |
| AT-CT-ST | 0.296 | 0.75 | 0.395 | 0.693 | (0.00,1.77) | 0 |
| CON-RT-CT | 0.147 | 0.369 | 0.397 | 0.691 | (0.00,0.87) | 0.01 |
| AT-RT-CT | 0.289 | 0.576 | 0.501 | 0.616 | (0.00,1.42) | 0 |
| RT-CT-ST | 0.06 | 0.937 | 0.064 | 0.949 | (0.00,1.90) | 0 |
| AT-RT-ST | 0.007 | 0.799 | 0.009 | 0.993 | (0.00,1.57) | 0 |

**Table S10. Global inconsistency model.**

| **chi2** | **Prob > chi2** |
| --- | --- |
| 2.21 | 0.9945 |

**Table S11. Node-splitting analysis.**

| **Side** | **Direct** | | **Indirect** | | **Difference** | |  |
| --- | --- | --- | --- | --- | --- | --- | --- |
|  | **Coef.** | **Std. Err.** | **Coef.** | **Std. Err.** | **Coef.** | **Std. Err.** | **P>\|z\|** |
| A B | -0.6520498 | 0.2680994 | -0.6333485 | 0.3990753 | -0.0187013 | 0.4813906 | 0.969 |
| A C | -0.2995682 | 0.4061993 | -0.6030279 | 0.5404358 | 0.3034597 | 0.6791383 | 0.655 |
| A D | -0.110953 | 0.3695645 | -0.4876935 | 0.4674194 | 0.3767406 | 0.595692 | 0.527 |
| A E | -1.354213 | 0.3753024 | -0.6224814 | 0.4769891 | -0.7317313 | 0.6097275 | 0.23 |
| B C | -0.2163186 | 0.6552247 | 0.4236263 | 0.4200608 | -0.6399448 | 0.7772993 | 0.41 |
| B D | 0.12676 | 0.5317729 | 0.540975 | 0.400723 | -0.4142151 | 0.6660123 | 0.534 |
| B E | -0.1404298 | 0.3690422 | -0.8971616 | 0.4749253 | 0.7567318 | 0.6023865 | 0.209 |
| B H | 0.3498501 | 0.6150989 | 0.9278981 | 0.8902324 | -0.5780481 | 1.082065 | 0.593 |
| C D | 0.0842161 | 0.5578615 | 0.2145891 | 0.5186989 | -0.1303729 | 0.7612259 | 0.864 |
| C H | 0.5560982 | 0.9423529 | 0.1590566 | 0.7034719 | 0.3970416 | 1.160047 | 0.732 |
| D H | 0.5135081 | 0.9235399 | -0.0466626 | 0.6760911 | 0.5601706 | 1.124683 | 0.618 |

**Table S12. Combined sensitivity-analysis league table using r = 0.25 and r = 0.75 for imputing change-score SDs**

| a. Sensitivity-analysis league table using r = 0.25 | | | | | | | |
| --- | --- | --- | --- | --- | --- | --- | --- |
| AT |  |  |  |  |  |  |  |
| -0.27 (-0.96,0.42) | RT |  |  |  |  |  |  |
| -0.40 (-1.02,0.21) | -0.13 (-0.86,0.59) | CT |  |  |  |  |  |
| 0.39 (-0.22,1.00) | 0.66 (-0.18,1.50) | 0.79 (0.01,1.58) | IT |  |  |  |  |
| -0.10 (-1.15,0.95) | 0.17 (-0.97,1.32) | 0.31 (-0.81,1.42) | -0.49 (-1.62,0.65) | WBVT |  |  |  |
| 0.26 (-1.65,2.17) | 0.53 (-1.43,2.49) | 0.67 (-1.28,2.61) | -0.13 (-2.08,1.83) | 0.36 (-1.73,2.45) | IHT |  |  |
| -0.53 (-1.56,0.50) | -0.26 (-1.42,0.89) | -0.13 (-1.25,0.99) | -0.92 (-2.10,0.26) | -0.43 (-1.88,1.01) | -0.79 (-2.94,1.36) | ST |  |
| -0.71 (-1.14,-0.28) | -0.44 (-1.06,0.18) | -0.30 (-0.87,0.26) | -1.10 (-1.70,-0.49) | -0.61 (-1.57,0.35) | -0.97 (-2.83,0.89) | -0.19 (-1.26,0.89) | CON |
| a. Sensitivity-analysis league table using r = 0.75 | | | | | | | |
| AT |  |  |  |  |  |  |  |
| -0.28 (-0.89,0.33) | RT |  |  |  |  |  |  |
| -0.45 (-1.01,0.10) | -0.17 (-0.83,0.48) | CT |  |  |  |  |  |
| 0.36 (-0.18,0.89) | 0.64 (-0.11,1.38) | 0.81 (0.11,1.52) | IT |  |  |  |  |
| -0.04 (-0.97,0.90) | 0.24 (-0.77,1.26) | 0.41 (-0.57,1.40) | -0.40 (-1.41,0.61) | WBVT |  |  |  |
| 0.26 (-1.31,1.83) | 0.54 (-1.08,2.16) | 0.71 (-0.89,2.32) | -0.10 (-1.71,1.52) | 0.30 (-1.44,2.04) | IHT |  |  |
| -0.57 (-1.41,0.28) | -0.28 (-1.24,0.67) | -0.11 (-1.04,0.82) | -0.92 (-1.91,0.06) | -0.53 (-1.76,0.71) | -0.83 (-2.59,0.94) | ST |  |
| -0.71 (-1.10,-0.32) | -0.43 (-0.98,0.12) | -0.26 (-0.76,0.24) | -1.07 (-1.61,-0.53) | -0.67 (-1.52,0.18) | -0.97 (-2.49,0.55) | -0.14 (-1.04,0.75) | CON |

Note: Values are WMDs with 95% CIs for cfPWV. Each cell represents the column-defining treatment minus the row-defining treatment. Negative WMD values favor the column-defining treatment, because lower cfPWV indicates greater improvement in central arterial stiffness; positive WMD values favor the row-defining treatment. AT, aerobic training; CON, control; CT, combined training; cfPWV, carotid-femoral pulse wave velocity; IHT, isometric handgrip training; IT, interval training; RT, resistance training; ST, stretching training; WBVT, whole-body vibration training.

**Fig. S6. Forest plots of eligible comparisons of outcome indicator.**

**Table S13. Sensitivity analysis of SUCRA rankings under alternative correlation coefficients for imputing change-score SDs**

| **Treatment** | **r = 0.25** | | | **r = 0.50** | | | **r = 0.75** | | |
| --- | --- | --- | --- | --- | --- | --- | --- | --- | --- |
|  | **SUCRA** | **PrBest (%)** | **Mean rank** | **SUCRA** | **PrBest (%)** | **Mean rank** | **SUCRA** | **PrBest (%)** | **Mean rank** |
| CON | 12.1 | 0 | 7.1 | 13.5 | 0 | 7.1 | 11.1 | 0 | 7.2 |
| AT | 65.8 | 2.5 | 3.4 | 63.6 | 1.5 | 3.5 | 66.5 | 2.2 | 3.3 |
| RT | 45.5 | 1.6 | 4.8 | 45.6 | 1.6 | 4.8 | 45.1 | 1.1 | 4.8 |
| CT | 35.1 | 0.5 | 5.5 | 33.7 | 0.3 | 5.6 | 31.1 | 0 | 5.8 |
| IT | 87.1 | 40.7 | 1.9 | 87.7 | 41.5 | 1.9 | 87.9 | 43 | 1.8 |
| WBVT | 56.7 | 11.2 | 4 | 59.4 | 12.6 | 3.8 | 61.1 | 12.1 | 3.7 |
| IHT | 67.1 | 40.9 | 3.3 | 69 | 40.6 | 3.2 | 70.5 | 40.5 | 3.1 |
| ST | 30.6 | 2.7 | 5.9 | 27.5 | 1.9 | 6.1 | 26.7 | 1.1 | 6.1 |

**Key assumptions of Network Meta-Analysis**

There are three key assumptions underpinning the validity of network meta-analysis (NMA): network connectivity, transitivity, and consistency.

1. Connectivity

Connectivity is a key assumption in NMA which if deemed insufficient (i.e., due to lack of direct comparators) can lead to low statistical power and misleading results. In our study, we assessed connectivity of the network at both treatment and agent levels visually and found no evidence of unconnectedness on either network (Figs. S7 and S8).

**Fig. S7. Treatment-level network.**

The first value indicates the specific intervention and the second one indicates the corresponding dose category (0, 300, 600, 900, 1200 or 1500 MET·min/week) used for visualization. AT: aerobic training; RT: resistance training; CT: combined training; IT: interval training; IHT: isometric handgrip training; ST: stretching training; WBVT: whole-body vibration training.

**Fig. S8. Agent-level network.**

2. Consistency

We carried out consistency analysis in the data through the comparison of consistent (i.e., network effect sizes) and unrelated mean effects (UME) models (i.e., pairwise effect sizes) of the network.

In practice, we checked whether deviance, the number of estimated parameters in the network, and the Deviance Information Criterion (DIC) indicators were similar for both models which would indicate a good fit. Comparison of these parameters indicated good consistency across models (Table S14).

**Table S14. Fit comparison between consistency and unrelated mean effects models.**

| **Model** | **pV** | **Residual deviance** | **DIC** |
| --- | --- | --- | --- |
| Consistency | 63.89 | 69.32 | 133.21 |
| UME | 64.42 | 72.26 | 136.68 |

DIC: Deviance Information Criterion; pV: effective number of parameters reported in the current JAGS output; UME: unrelated mean effects. Similar values of DIC and residual deviance between the consistency and UME models indicate acceptable consistency of the network.

3. Clinical and methodological comparability of potential effect modifiers (transitivity)
We assessed the plausibility of transitivity qualitatively by examining the distributions of key potential effect modifiers across the treatment nodes represented in the network, including population category, age, sex distribution, BMI, baseline cfPWV, trial duration, intervention frequency, exercise modality, and weekly exercise dose. These characteristics are summarized in Table S15. Because transitivity cannot be tested directly with a single statistical test, this assumption was judged descriptively, whereas statistical consistency was evaluated separately by comparing direct and indirect evidence using node-splitting analysis and by comparing the consistency and unrelated mean effects models. Fig. S9 presents the node-splitting density plots used to evaluate agreement between direct and indirect evidence within the network.

**Table S15. Distribution of potential effect modifiers across treatment nodes.**

| **Node** | **Studies/arms( n)** | **Population categories** | **Age(years)** | **Male(%)** | **BMI(kg/m²)** | **Baseline cfPWV(m/s)** | **Duration(weeks)** | **Weekly dose, MET·min/week** |
| --- | --- | --- | --- | --- | --- | --- | --- | --- |
| CON | 28/28 | HTN; MetS; OB; OW; Older adults; PMW; T2D | 57 (21–71) | 51 (0–100) | 31.13 (21.19–36) | 8.35 (5.8–15.98) | NA | 0 (0–0) |
| AT | 19/20 | HTN; MetS; OB; Older adults; T1D; T2D | 58.5 (27–73) | 40.85 (0–100) | 30.12 (22.2–38) | 8.62 (6.1–13.4) | 10 (6–52) | 765 (270–1080) |
| RT | 6/6 | HTN; MetS; OB; OW | 48.5 (21.5–52) | 23.1 (0–100) | 30.5 (26.8–34.19) | 7.45 (6.64–11.09) | 10.5 (3–36) | 536.25 (367.5–720) |
| CT | 10/13 | HTN; OB; OW; Older adults; T2D | 57 (41.8–70) | 49 (0–100) | 29.4 (23.85–35) | 9.2 (6.8–13.3) | 16 (8–52) | 744.3 (270–1147.5) |
| IT | 8/8 | HTN; OB; OW; Older adults; T1D; T2D | 48.15 (24.9–72) | 56.5 (0–100) | 31.25 (22.2–37.5) | 7.46 (6.1–16.29) | 12 (6–20) | 534.75 (405–1530) |
| WBVT | 3/3 | HTN; OB; PMW | 55.5 (20–57) | 0 (0–0) | 33.6 (30.7–34.2) | 12.4 (7.8–13.4) | 12 (6–12) | 189.5 (154–225) |
| IHT | 1/1 | HTN | 60.12 (60.12–60.12) | 30 (30–30) | 30.58 (30.58–30.58) | 9.19 (9.19–9.19) | 12 (12–12) | 124 (124–124) |
| ST | 3/3 | OW; Older adults | 66.1 (54–67.8) | 47 (30–50) | 26.8 (25.7–28.2) | 9.95 (8.99–11.33) | 52 (13–52) | 310 (241.5–316) |

Note: Values are summarized at the arm level and presented as median (range), unless otherwise indicated. Studies/arms refers to the number of unique studies and intervention/control arms contributing to each node. Baseline cfPWV was summarized using available arm-level baseline values; arms with missing baseline cfPWV values were excluded from this descriptive summary only. Weekly dose was summarized using exact MET·min/week values from Table S6; missing exact dose values were excluded from the dose summary. AT, aerobic training; BMI, body mass index; cfPWV, carotid-femoral pulse wave velocity; CON, control; CT, combined training; HTN, hypertension; IHT, isometric handgrip training; IT, interval training; MetS, metabolic syndrome; OB, obesity; OW, overweight; PMW, postmenopausal women; RT, resistance training; ST, stretching training; T1D, type 1 diabetes; T2D, type 2 diabetes; WBVT, whole-body vibration training.

**Fig. S9. Node-splitting density plots.**

Note. Model selection and fit diagnostics.

A meta-analysis (i.e., a "split" NMA) of the different doses of physical activity as separate and unrelated treatments were performed. This step helps determine which function is more appropriate for the data and should be used in a model-based network meta-analysis (MBNMA).

1. Model selection

**Table S16. Model fit comparison for dose-response network meta-analysis.**

| **Model** | **DIC** | **Residual deviance** | **pD** |
| --- | --- | --- | --- |
| Emax (common treatment effects) | 340.51 | 342.88 | 41.08 |
| Restricted cubic spline | 231.35 | 231.63 | 44.81 |
| Quadratic function (random treatment effects) | 87.06 | 68.45 | 62.84 |

The quadratic random-effects model provided the best fit, with the lowest DIC (87.06) and residual deviance (68.45). The restricted cubic spline and Emax models showed poorer fit, with DIC values of 231.35 and 340.51, respectively. Therefore, the quadratic model was retained for subsequent dose-response analyses.

2. Deviance check

In addition to the model fit index, a deviation plot showing the contribution of each data point to the residuals can also help to confirm the robustness of the model selection. The contribution of each data point to the the posterior mean residual deviance for each data point should be close to 1, which indicates a good model fit. Deviation plots for overall (Fig. S10) treatment effects (Fig. S11) confirm the robustness of our model selection (i.e., deviations <1.5).

**Fig. S10. Deviance plot at the overall exercise level.**

**Fig. S11. Deviance plots at the treatment level.**

3.Degree of fit test

In addition, we also plotted the fit further to assess the degree of fit of the model. The fit values are plotted as connecting lines, and the observations in the original dataset are plotted as points. These plots can determine if the model fits the data well for different exercises and doses of the dose-response function.

**Fig. S12. Fit plots at the overall exercise level.**

**Fig. S13. Fit plots at the agent level.**

Note. Exercise dose and carotid-femoral pulse wave velocity dose-response relationships.

**Table S17. MCMC convergence diagnostics for the Bayesian dose-response models**

| **Model** | **Chains** | **Iterations per chain** | **Burn-in** | **Thinning interval** | **Posterior samples per chain** | **Total posterior samples** | **Monitored parameters, n** | **Trace-plot parameters, n** | **PSRF/R-hat point estimate range** | **PSRF/R-hat upper CI range** | **Convergence judgment** |
| --- | --- | --- | --- | --- | --- | --- | --- | --- | --- | --- | --- |
| Overall exercise dose-response model | 4 | 40000 | 20000 | 10 | 2000 | 8000 | 5 | 3 | 0.99994–1.00039 | 1.00032–1.00171 | Adequate |
| Exercise-type-specific dose-response model | 4 | 40000 | 20000 | 10 | 2000 | 8000 | 17 | 15 | 0.99987–1.00059 | 0.99993–1.00167 | Adequate |

Notes: PSRF, potential scale reduction factor; R-hat, Gelman–Rubin convergence diagnostic; CI, confidence interval. “Monitored parameters” refers to the total number of parameters assessed in the convergence diagnostics, whereas “Trace-plot parameters” refers to the parameters displayed in the supplementary trace-plot figures.

**Fig. S14. Trace plots for the overall exercise dose-response model.**

Trace plots for the overall exercise dose-response model and the exercise-type-specific dose-response model. Trace plots were used to visually assess Markov chain Monte Carlo convergence for the Bayesian dose-response models. Adequate convergence was indicated by stable, well-mixed chains without clear trends or separation between chains.

**Fig. S15. Trace plots for the exercise-type-specific dose-response model.**

**Fig. S16. Overall exercise dose and carotid-femoral pulse wave velocity dose-response relationship.**

**Fig. S17. Exercise-type-specific dose-response relationships between exercise dose and carotid-femoral pulse wave velocity.**

Dose-response relationship between exercise dose and cfPWV. The green shaded area in the figure indicates the original study dataset; the darker the color, the larger the amount of data.

**Fig. S18. Ranking distributions of intervention-dose combinations.**

The first value indicates the specific intervention and the second one is the corresponding dose of that intervention (MET·min/week).

**Fig. S19. Summary of study limitations of the included studies.**

Line colours indicate the majority RoB 2 overall judgment among studies contributing to each direct comparison: low risk of bias (green), some concerns (yellow), and high risk of bias (red). When judgments were tied within a comparison, the more conservative category was assigned in the order high risk > some concerns > low risk. Node size is proportional to the total sample size for each intervention, and line width is proportional to the number of studies contributing to each direct comparison.

**Fig. S20. Contribution of risk-of-bias comparisons to each network estimate.**

Based on the RoB 2 assessment for each direct comparison and the contribution of direct comparisons to each network estimate, the bar plot shows the percentage contribution of low-risk, some-concerns, and high-risk evidence to each network estimate.

**Table S18. Certainty of evidence for carotid-femoral pulse wave velocity.**

| Comparison | Number of studies | Study design | Risk of bias | Inconsistency / incoherence | Indirectness | Imprecision | Other considerations | Intervention | Control group / Other intervention | Mean difference (95% CI) | Certainty | Importance |
| --- | --- | --- | --- | --- | --- | --- | --- | --- | --- | --- | --- | --- |
| Overall exercise compared to Control (CON) for cfPWV (duration: 3 to 52 weeks) | 27 RCTs | RCT | Serious a | Serious b | Not serious c | Not serious | Publication bias not detected d | 975 | 697 | -0.65 (-1.13, -0.18) | ⨁⨁◯◯ LOW | CRITICAL |
| Aerobic training (AT) compared to Control (CON) for cfPWV (duration: 8 to 24 weeks) | 11 RCTs | RCT | Serious a | Serious e | Not serious c | Not serious | Publication bias not detected d | 448 | 329 | -0.78 (-1.37, -0.19) | ⨁⨁◯◯ LOW | CRITICAL |
| Resistance training (RT) compared to Control (CON) for cfPWV (duration: 3 to 12 weeks) | 4 RCTs | RCT | Serious a | Not serious | Not serious c | Serious k | - | 60 | 42 | -0.27 (-0.71, 0.17) | ⨁⨁◯◯ LOW | CRITICAL |
| Combined training (CT) compared to Control (CON) for cfPWV (duration: 8 to 52 weeks) | 7 RCTs | RCT | Serious a | Not serious | Not serious c | Serious k | - | 259 | 187 | -0.21 (-0.65, 0.22) | ⨁⨁◯◯ LOW | CRITICAL |
| Interval training (IT) compared to Control (CON) for cfPWV (duration: 12 weeks) | 3 RCTs | RCT | Not serious f | Serious l | Not serious c | Serious k | - | 94 | 94 | -1.45 (-4.18, 1.27) | ⨁⨁◯◯ LOW | CRITICAL |
| Whole-body vibration training (WBVT) compared to Control (CON) for cfPWV (duration: 6 to 12 weeks) | 3 RCTs | RCT | Not serious f | Not serious | Not serious c | Serious h | Sparse evidence | 62 | 37 | -0.40 (-0.64, -0.16) | ⨁⨁⨁◯ MODERATE | CRITICAL |
| Isometric handgrip training (IHT) compared to Control (CON) for cfPWV (duration: 12 weeks) | 1 RCT | RCT | Not serious f | Not serious m | Not serious c | Very serious j | Sparse evidence | 32 | 16 | -0.97 (-2.12, 0.18) | ⨁⨁◯◯ LOW | CRITICAL |
| Stretching training (ST) compared to Control (CON) for cfPWV (network estimate; no direct head-to-head trial) | 0 direct RCTs | RCT | Serious a | Not serious g | Serious i | Very serious j | Indirect estimate only; sparse evidence | NA | NA | -0.11 (-1.14, 0.92) | ⨁◯◯◯ VERY LOW | CRITICAL |
| Aerobic training (AT) compared to Resistance training (RT) for cfPWV (duration: 36 weeks) | 1 RCT | RCT | Serious a | Not serious m | Not serious c | Very serious j | Sparse direct evidence | 50 | 30 | -0.24 (-0.93, 0.46) | ⨁◯◯◯ VERY LOW | IMPORTANT |
| Aerobic training (AT) compared to Combined training (CT) for cfPWV (duration: 8 to 36 weeks) | 3 RCTs | RCT | Serious a | Not serious g | Not serious c | Serious k | - | 107 | 83 | -0.39 (-1.01, 0.23) | ⨁⨁◯◯ LOW | IMPORTANT |
| Aerobic training (AT) compared to Interval training (IT) for cfPWV (duration: 6 to 20 weeks) | 5 RCTs | RCT | Not serious f | Not serious g | Not serious c | Serious k | - | 67 | 62 | 0.42 (-0.15, 1.00) | ⨁⨁⨁◯ MODERATE | IMPORTANT |
| Aerobic training (AT) compared to Whole-body vibration training (WBVT) for cfPWV (network estimate; no direct head-to-head trial) | 0 direct RCTs | RCT | Not serious f | Not serious g | Serious i | Very serious j | Indirect estimate only; sparse evidence | NA | NA | -0.01 (-1.05, 1.03) | ⨁◯◯◯ VERY LOW | IMPORTANT |
| Aerobic training (AT) compared to Isometric handgrip training (IHT) for cfPWV (network estimate; no direct head-to-head trial) | 0 direct RCTs | RCT | Not serious f | Not serious g | Serious i | Very serious j | Indirect estimate only; sparse evidence | NA | NA | 0.32 (-1.50, 2.14) | ⨁◯◯◯ VERY LOW | IMPORTANT |
| Aerobic training (AT) compared to Stretching training (ST) for cfPWV (duration: 52 weeks) | 2 RCTs | RCT | Serious a | Not serious g | Not serious c | Serious k | - | 46 | 55 | -0.54 (-1.52, 0.45) | ⨁⨁◯◯ LOW | IMPORTANT |
| Resistance training (RT) compared to Combined training (CT) for cfPWV (duration: 13 to 36 weeks) | 2 RCTs | RCT | Serious a | Not serious g | Not serious c | Serious k | - | 43 | 42 | -0.15 (-0.89, 0.58) | ⨁⨁◯◯ LOW | IMPORTANT |
| Resistance training (RT) compared to Interval training (IT) for cfPWV (network estimate; no direct head-to-head trial) | 0 direct RCTs | RCT | Serious a | Not serious g | Serious i | Serious k | Indirect estimate only | NA | NA | 0.66 (-0.16, 1.49) | ⨁◯◯◯ VERY LOW | IMPORTANT |
| Resistance training (RT) compared to Whole-body vibration training (WBVT) for cfPWV (network estimate; no direct head-to-head trial) | 0 direct RCTs | RCT | Serious a | Not serious g | Serious i | Very serious j | Indirect estimate only; sparse evidence | NA | NA | 0.23 (-0.91, 1.37) | ⨁◯◯◯ VERY LOW | IMPORTANT |
| Resistance training (RT) compared to Isometric handgrip training (IHT) for cfPWV (network estimate; no direct head-to-head trial) | 0 direct RCTs | RCT | Serious a | Not serious g | Serious i | Very serious j | Indirect estimate only; sparse evidence | NA | NA | 0.56 (-1.32, 2.44) | ⨁◯◯◯ VERY LOW | IMPORTANT |
| Resistance training (RT) compared to Stretching training (ST) for cfPWV (duration: 13 weeks) | 1 RCT | RCT | Serious a | Not serious m | Not serious c | Very serious j | Sparse direct evidence | 13 | 12 | -0.30 (-1.41, 0.81) | ⨁◯◯◯ VERY LOW | IMPORTANT |
| Combined training (CT) compared to Interval training (IT) for cfPWV (network estimate; no direct head-to-head trial) | 0 direct RCTs | RCT | Serious a | Not serious g | Serious i | Serious k | Indirect estimate only; no direct head-to-head trial | NA | NA | 0.82 (0.04, 1.59) | ⨁◯◯◯ VERY LOW | IMPORTANT |
| Combined training (CT) compared to Whole-body vibration training (WBVT) for cfPWV (network estimate; no direct head-to-head trial) | 0 direct RCTs | RCT | Serious a | Not serious g | Serious i | Very serious j | Indirect estimate only; sparse evidence | NA | NA | 0.38 (-0.72, 1.49) | ⨁◯◯◯ VERY LOW | IMPORTANT |
| Combined training (CT) compared to Isometric handgrip training (IHT) for cfPWV (network estimate; no direct head-to-head trial) | 0 direct RCTs | RCT | Serious a | Not serious g | Serious i | Very serious j | Indirect estimate only; sparse evidence | NA | NA | 0.71 (-1.14, 2.57) | ⨁◯◯◯ VERY LOW | IMPORTANT |
| Combined training (CT) compared to Stretching training (ST) for cfPWV (duration: 13 weeks) | 1 RCT (1 direct contrast) | RCT | Serious a | Not serious m | Not serious c | Very serious j | Sparse direct evidence | 12 | 12 | -0.15 (-1.22, 0.93) | ⨁◯◯◯ VERY LOW | IMPORTANT |
| Interval training (IT) compared to Whole-body vibration training (WBVT) for cfPWV (network estimate; no direct head-to-head trial) | 0 direct RCTs | RCT | Not serious f | Not serious g | Serious i | Very serious j | Indirect estimate only; sparse evidence | NA | NA | -0.43 (-1.55, 0.68) | ⨁◯◯◯ VERY LOW | IMPORTANT |
| Interval training (IT) compared to Isometric handgrip training (IHT) for cfPWV (network estimate; no direct head-to-head trial) | 0 direct RCTs | RCT | Not serious f | Not serious g | Serious i | Very serious j | Indirect estimate only; sparse evidence | NA | NA | -0.10 (-1.97, 1.76) | ⨁◯◯◯ VERY LOW | IMPORTANT |
| Interval training (IT) compared to Stretching training (ST) for cfPWV (network estimate; no direct head-to-head trial) | 0 direct RCTs | RCT | Serious a | Not serious g | Serious i | Very serious j | Indirect estimate only; sparse evidence | NA | NA | -0.96 (-2.08, 0.16) | ⨁◯◯◯ VERY LOW | IMPORTANT |
| Whole-body vibration training (WBVT) compared to Isometric handgrip training (IHT) for cfPWV (network estimate; no direct head-to-head trial) | 0 direct RCTs | RCT | Not serious f | Not serious g | Serious i | Very serious j | Indirect estimate only; sparse evidence | NA | NA | 0.33 (-1.68, 2.34) | ⨁◯◯◯ VERY LOW | IMPORTANT |
| Whole-body vibration training (WBVT) compared to Stretching training (ST) for cfPWV (network estimate; no direct head-to-head trial) | 0 direct RCTs | RCT | Serious a | Not serious g | Serious i | Very serious j | Indirect estimate only; sparse evidence | NA | NA | -0.53 (-1.93, 0.88) | ⨁◯◯◯ VERY LOW | IMPORTANT |
| Isometric handgrip training (IHT) compared to Stretching training (ST) for cfPWV (network estimate; no direct head-to-head trial) | 0 direct RCTs | RCT | Serious a | Not serious g | Serious i | Very serious j | Indirect estimate only; sparse evidence | NA | NA | -0.86 (-2.91, 1.19) | ⨁◯◯◯ VERY LOW | IMPORTANT |

Note: Values are mean differences for the first intervention relative to the second intervention; negative values indicate lower cfPWV in the first intervention.

Exercise-versus-control comparisons were graded against the direct pairwise estimates, whereas exercise-versus-exercise comparisons were graded against the network estimates. Where multi-arm trials contributed more than one exercise-versus-control comparison in the pairwise meta-analysis, the shared control group was split evenly across comparisons to avoid double-counting.

The “Intervention” and “Control group/Other intervention” columns summarize the direct evidence base contributing to each comparison and are reported as NA when no head-to-head trial was available. CI, confidence interval; cfPWV, carotid–femoral pulse wave velocity; CON, control; RCT, randomized controlled trial; RT, resistance training; CT, combined training; IT, interval training; WBVT, whole-body vibration training; IHT, isometric handgrip training; ST, stretching training.

a. Downgraded one level for study limitations because the contributing evidence included some-concerns and/or high-risk RoB 2 judgments, or because the corresponding network estimate was not predominantly informed by low-risk direct evidence in the contribution plot.

b. Downgraded one level for inconsistency because the overall pairwise exercise-versus-control comparison showed considerable heterogeneity (I² = 94.4%).

c. Not downgraded for indirectness because the review question, target population, intervention framework, comparator framework, and primary outcome (cfPWV) were directly aligned.

d. Not downgraded for publication bias because Egger’s test for the overall pairwise analysis was not significant and the comparison-adjusted funnel plot showed no obvious asymmetry.

e. Downgraded one level for inconsistency because the direct AT-versus-control comparison showed considerable heterogeneity (I² = 87.6%).

f. Not downgraded for study limitations because the corresponding direct comparison was predominantly rated as low risk in RoB 2, or because the network contribution profile was mainly informed by low-risk direct evidence.

g. Not downgraded for inconsistency or incoherence at the network level because the loop-specific heterogeneity estimates, global inconsistency model, and node-splitting analysis did not indicate statistically significant inconsistency.

h. Downgraded one level for imprecision because the comparison was based on a small number of studies and/or limited direct evidence.

i. Downgraded one level for indirectness because the estimate was based on indirect network evidence only, with no direct head-to-head comparison.

j. Downgraded two levels for imprecision because the confidence interval was very wide and the comparison was supported by sparse evidence.

k. Downgraded one level for imprecision because the confidence interval crossed the null and included both potential benefit and no clear effect.

l. Downgraded one level for inconsistency because the direct IT-versus-control comparison showed considerable heterogeneity (I² = 98.2%).

m. Inconsistency could not be meaningfully assessed because only one direct study was available; sparse evidence was therefore reflected under imprecision and other considerations rather than inconsistency.
